# Are Conventional Type 1 Dendritic Cells Critical for Protective Antitumor Immunity and How?

**DOI:** 10.3389/fimmu.2019.00009

**Published:** 2019-02-12

**Authors:** Jean-Charles Cancel, Karine Crozat, Marc Dalod, Raphaël Mattiuz

**Affiliations:** CNRS, INSERM, Centre d'Immunologie de Marseille-Luminy, Turing Center for Living Systems, Aix Marseille University, Marseille, France

**Keywords:** conventional type 1 dendritic cells, tumor, type I IFN, CD8^+^ T cells, NK cells, immunotherapy, cancer immunosurveillance, clinical trials

## Abstract

Dendritic cells (DCs) are endowed with a unique potency to prime T cells, as well as to orchestrate their expansion, functional polarization and effector activity in non-lymphoid tissues or in their draining lymph nodes. The concept of harnessing DC immunogenicity to induce protective responses in cancer patients was put forward about 25 years ago and has led to a multitude of DC-based vaccine trials. However, until very recently, objective clinical responses were below expectations. Conventional type 1 DCs (cDC1) excel in the activation of cytotoxic lymphocytes including CD8^+^ T cells (CTLs), natural killer (NK) cells, and NKT cells, which are all critical effector cell types in antitumor immunity. Efforts to investigate whether cDC1 might orchestrate immune defenses against cancer are ongoing, thanks to the recent blossoming of tools allowing their manipulation *in vivo*. Here we are reporting on these studies. We discuss the mouse models used to genetically deplete or manipulate cDC1, and their main caveats. We present current knowledge on the role of cDC1 in the spontaneous immune rejection of tumors engrafted in syngeneic mouse recipients, as a surrogate model to cancer immunosurveillance, and how this process is promoted by type I interferon (IFN-I) effects on cDC1. We also discuss cDC1 implication in promoting the protective effects of immunotherapies in mouse preclinical models, especially for adoptive cell transfer (ACT) and immune checkpoint blockers (ICB). We elaborate on how to improve this process by *in vivo* reprogramming of certain cDC1 functions with off-the-shelf compounds. We also summarize and discuss basic research and clinical data supporting the hypothesis that the protective antitumor functions of cDC1 inferred from mouse preclinical models are conserved in humans. This analysis supports potential applicability to cancer patients of the cDC1-targeting adjuvant immunotherapies showing promising results in mouse models. Nonetheless, further investigations on cDC1 and their implications in anti-cancer mechanisms are needed to determine whether they are the missing key that will ultimately help switching cold tumors into therapeutically responsive hot tumors, and how precisely they mediate their protective effects.

## Introduction

Immune responses against cancer are sculpted by the tumor microenvironment, including its composition in terms of cell types and their physiological states. Indeed, tumors escape host immune defenses not only through decreasing their intrinsic immunogenicity but also by shaping a specific immunosuppressive microenvironment (1, 2). Exogenous factors such as the microbiota and its metabolites also modulate the tumor microenvironment and hence antitumor immune responses (3). According to their degree of infiltration by immune cells and to their capacity to activate antitumor immune responses, tumors have been classified as immunologically “Hot” or “Cold.” “Hot” tumors are immunogenic, T cell-inflamed, and efficiently rejected by the immune system. They are characterized by the presence of activated CD8^+^ cytotoxic T lymphocytes (CTLs), by the expression of T cell-attracting chemokines, and by a type I interferon (IFN-I) transcriptional signature (4). “Cold” tumors lack T cell infiltration, which is correlated with an absence of IFN-I signature and with a poor chemokine production (4). They are ignored by the immune system due to their poor immunogenicity and are very poorly responsive to immunotherapies. We propose to refine this bimodal classification through the addition of two other tumor states, which we called “Icy” and “Warm.” We define as “Icy” the tumors that develop potent, active, mechanisms to prevent immune recognition and T cell activation, by inducing a highly immunosuppressive microenvironment very early on during their development. Hence, “Icy” tumors are even more refractory to immune control than “Cold” tumors. “Warm” tumors present an intermediate level of infiltration by “exhausted” CTLs, which have been functionally paralyzed by the local immunosuppressive environment that has been progressively shaped during tumor development. The exhaustion of CTLs is at least in part due to engagement of their immune checkpoint receptors by ligands expressed by the tumor cells themselves or by infiltrating antigen (Ag)-presenting cells. “Warm” tumors are more prone to be controlled by immune checkpoint blockade (ICB) treatments. These monoclonal antibody (mAb)-based immunotherapies have revolutionized cancer patient care, by significantly increasing not only overall survival rates but also very long-term remissions for tumor types previously difficult to treat. Despite this major advance, the majority of patients with difficult-to-treat cancers do not respond to ICB. To overcome this issue, it is critical to find additional means of manipulating the microenvironment of the “Cold” or “Warm” tumors unresponsive to ICB, in order to convert them into “Hot” tumors. It should be possible to achieve this by combining ICB with adjuvant immunotherapies able to counteract the other immune escape mechanisms established by these tumors, in order to (i) trigger *de novo* or enhance T cell infiltration, (ii) enhance cross-presentation of tumor-associated Ag, and (iii) promote a better induction or reactivation of CTL effector functions.

Dendritic cells (DCs) are the most potent Ag-presenting cells, with a unique efficacy for priming naïve T cells and inducing their functional polarization. They are more generally in charge of orchestrating the expansion and functions of T and natural killer (NK) cells in lymphoid and non-lymphoid tissues. Many clinical trials have been performed over the last 25 years to attempt harnessing DC functions for boosting protective antitumor CTL responses in cancer patients (5). Up to now, the results have been disappointingly far below expectations. These failures occurred at least in part because of the almost exclusive use of monocyte-derived DCs (MoDCs) for ACT in cancer patients. Indeed, later advancement of our basic understanding of the heterogeneity and functional plasticity of DCs suggested that other types than MoDCs should be better suited for this purpose (6–8). A relatively recent consensus has emerged on a universal and simplified classification of DC types both in mice and in humans, based on their ontogeny, gene expression programs, phenotype, functions and localization (9, 10). Five major types of DCs can be distinguished: plasmacytoid DC (pDCs), type 1 conventional DCs (cDC1), type 2 cDCs (cDC2), Langerhans cells and MoDCs. In mice, cDC1 encompass both the lymphoid tissue-resident CD8α^+^ cDCs as well as the CD103^+^CD11b^−^ cDCs that reside in the parenchyma of non-lymphoid tissues and, once matured upon activation, can migrate to the draining lymph nodes. In humans, cDC1 correspond to the CD141 (BDCA3)^high^ CD11b^−/low^ cDCs. Both mouse and human cDC1 express specifically the chemokine receptor XCR1 and selectively the C-type lectin endocytic receptor CLEC9A (11). cDC1 can directly enter tissues from the blood, or differentiate locally from a dedicated progenitor, the pre-cDC1 that has been characterized both in the mouse and the human (12, 13). Mouse cDC2 correspond to the CD11b^+^ cDCs, and human cDC2 to the CD1c (BDCA1)^high^ CD11b^+/high^ cDCs. For a very long time, MoDCs were the only DC type that could be produced *in vitro*, in high numbers and under clinical-grade conditions (5, 6, 8). They were therefore used for most immunotherapeutic clinical trials based on adoptive cell transfer (ACT) of *in vitro* derived autologous DCs. However, MoDCs strikingly differ from cDC1 and cDC2 that are the major types of DCs residing in secondary lymphoid organs and orchestrating immune responses *in vivo* (14–16). For example, MoDCs do not migrate efficiently to lymph nodes and are particularly prone to develop immunosuppressive functions, whereas cDC1 excel in the activation of CTLs, which are critical effector cell types for antitumor immunity (17). Thus, major efforts have been conducted in the last 10 years to investigate whether cDC1 might be critical for defense against cancer, and how. Here, we are reporting on studies addressing this issue in mice, under experimental conditions of spontaneous immune rejection of tumor grafts in syngeneic recipients, or in preclinical models of immunotherapies. We also summarize human studies that mined large datasets of tumor gene expression profiles to investigate correlations between clinical outcome and digital deconvolution of the tumor immune infiltrate. We discuss how the knowledge generated by these studies can instruct innovative immunotherapeutic strategies to harness cDC1 functions for the benefits of cancer patients.

## No Currently Available Mutant Mouse Model is Specifically Targeting Only cDC1 *in vivo*

To determine whether and how a given type of immune cells plays a non-redundant role in antitumor immunity *in vivo*, it should be specifically and efficiently manipulated in mice. Different mutant mouse models have been generated to either deplete DCs, or inactivate candidate genes in DCs, as recently reviewed (18, 19). Here, we will specifically discuss the use of mutant mouse models to investigate the functions of cDC1 or their molecular regulation (Table 1) (14, 20–47). Mouse models expressing the Cre DNA recombinase under the control of the promoter of a gene selectively expressed in DCs have been generated to enable conditional deletion of candidate floxed genes in the targeted cells (e.g., *Itgax*-*Cre* targeting CD11c^+^ cells and *Xcr1*-*Cre* targeting cDC1). Constitutive depletion models have been generated using two types of strategies. The first corresponds to the knock-out of a transcription factor shown to be crucial selectively for the development/homeostasis of cDCs (Zbtb46) or cDC1 (Batf3) (Table 1). The second consists in ectopic expression of the active subunit of the diphtheria toxin (*DTA*) selectively in DCs (e.g., *Xcr1-Cre*;*Rosa26-LSL-DTA* mice for cDC1, Table 1). Conditional depletion can be achieved upon diphtheria toxin administration in mutant animals engineered for ectopic expression of the gene encoding the human diphtheria toxin receptor (*hDTR*) selectively in DCs (e.g., *Karma*-*hDTR* or *Xcr1-hDTR* mice for cDC1).

**Table 1:** Mouse models to deplete DCs, cDCs or cDC1 *in vivo*.

**Mouse strain**	**Depleted cells**	**Gene also expressed in**	**Remarks**	**Expression profile references**
CD11c-hDTR*	cDCs (pDCs?)	NK cells Effector/memory CTL Monocytes, macrophages Plasmablasts IELs	Off-target transgene expression leading to death upon multiple DT injections (Requiring to perform BM chimeras for prolonged depletion)	**(****20****)** (21–27) ImmGen Consortium
CD11c.DOG*	DCs	NK cells Effector/memory CTL Monocytes, Macrophages Plasmablasts IELs	hDTR expression only in CD11c^+^ cells Prolonged DC depletion possible upon multiple DT injections Ovalbumin protein is expressed in DCs, resulting in extensive OT-I and OT-II proliferation after transfer	**(****28****)** (20–27) ImmGen Consortium
CD205-hDTR	cDC1 and LCs	Cortical thymic epithelium Tumor MoDC and cDC2	Death induced by DT injection Use of BM chimeras required to avoid death consecutive to depletion of radioresistant CD205^+^ cells	**(****29****)** (30, 31)
Zbtb46-hDTR*	cDC1 and cDC2	Endothelial cells Committed erythroid progenitors	Death induced by a single DT injection Use of BM chimeras required	**(****32****)** (14, 33)
Zbtb46-LSL-hDTR*	cDC1 and cDC2	Endothelial cells Committed erythroid progenitors	Allows prolonged cDC depletion upon multiple DT injections (Need to cross with a Cre strain)	**(****34****)** (14, 32, 33)
*Batf3^−/−^*	cDC1	cDC2 Eff (Th1) CD4^+^ T cells Other T cells?	cDC1 depletion is effective in *129/SvEv* mice but less in *C57BL/6* animals Intracellular pathogens infections or IL-12 injection restore cDC1 development Higher differentiation in Treg of *Batf*3^−/−^ CD4^+^ T cells	**(****35****)** (14, 36–40)
Clec9a-hDTR*	cDC1	pDCs	Half of the pDCs are depleted	**(****41****)**, (42)
Karma-hDTR*	cDC1	Skin and PC Mast cells	Mast cells are targeted in addition to cDC1	**(****43****)**, (42)
XCR1-hDTR* (Kaisho)	cDC1		Deletion of the endogenous *Xcr1* gene Requiring the use of heterozygous mice	**(****44****)**, (42)
XCR1-hDTR** (Dalod)	cDC1		Fate mapping of a minute proportion of CD4^+^ T cells	(14, 43, 45), **(****42****)**
XCR1-DTA*** (Kaisho)	cDC1		Deletion of the endogenous *Xcr1* gene Requiring the use of heterozygous mice	(14, 43) **(****46****)**, (45)
XCR1-DTA*** (Dalod)	cDC1		Fate mapping of a minute proportion of CD4^+^ T cells	(14, 43, 45), **(****42****)**

*Bold: First publication*.

* *Mouse models expressing the Cre DNA recombinase under the same gene promoter have been generated*.

** *Xcr1-Cre;Rosa26-LSL-hDTR mice*;

*** *Xcr1-Cre;Rosa26-LSL-DTA mice*.

One major caveat of using CD11c for targeting DCs is that the gene encoding this molecule, *Itgax*, is expressed by other immune cell types, including some that play critical roles in anti-tumor immunity, such as NK cells, effector memory CTLs, intraepithelial lymphocytes (IELs), plasmablasts, and subsets of monocytes or macrophages (32). Knock-in within the *Zbtb46* gene has been used to target all cDCs. However, this gene is also expressed by endothelial cells and committed erythroid progenitors (14, 32–34). Since angiogenesis critically affects solid tumor development, experiments should be performed using bone marrow chimera mice generated by engrafting mutant bone marrow cells into a wild type (WT) recipient animal. *Batf3*^−/−^ mice have been the most frequently used model to investigate whether cDC1 play a critical role in physiological processes. However, even in this model, complementary strategies are needed before drawing final conclusions, because *Batf3* is also expressed in cDC2 and effector CD4^+^ T cells, and because it represses *Foxp3* expression in CD4^+^ T cells leading to increased numbers of regulatory T cells (Treg) in knock-out mice (35, 36). In addition, the impact of *Batf3* inactivation on cDC1 homeostasis is less efficient in the *C57BL6/J* genetic background than in the *129svEv* one. Under inflammatory settings, the knock-out of *Batf3* can be compensated for cDC1 development, by the induction in DC precursors of the paralog genes *Batf* and *Batf2* (37–39). We have engineered mutant mouse models for cDC1 targeting based on the knock-in of Cre (42) or hDTR (43) into the *Gpr141b* (alias *A530099j19rik* or *Karma)* gene, but these models also target mast cells (42). Finally, the *Xcr1* gene was targeted to generate mutant mouse models for specific, conditional or constitutive, cDC1 depletion, as well as for their genetic manipulation (42, 44, 46). The *Xcr1* gene is preserved in our models (42). In contrast, it is knocked-out in the other ones (46); hence, only heterozygous mice should be used for these models in order to avoid possible phenotypic effects due to a complete XCR1 deficiency. Besides cDC1, only a minute proportion of CD4^+^ T cells are targeted in *Xcr1*-*Cre* mice (42). Although still imperfect, the mutant mouse models based on the manipulation of the *Xcr1* gene are the best to target cDC1 *in vivo*. In conclusion, none of the mutant mouse models used to date for cDC1 targeting are entirely specific and efficient, but some are better suited than others for this purpose. In any case, it is always important to use complementary methods to ensure that the phenotypes observed are only or mostly due to the manipulation of cDC1. For example, depleted mice should be replenished with wild-type cDC1 if possible. Alternately, results should be confirmed in other mutant models also targeting cDC1 but no other cell types in common.

## The Role of cDC1 in Cancer immunosurveillance has not yet been Investigated

Cancer development is a multistep process consisting in the accumulation of genetic mutations within a cell leading to increased or deregulated proliferation and survival, with clonal selection of neoplastic progeny (48). There is a strong contribution of the host immune responses in this dynamical process of tumor selection, which has been described as the three E of cancer immunoediting: Elimination, Equilibrium and Escape of cancer cells (49). A failure of the immune system to eliminate all transformed cells early during their development is followed by an equilibrium state during which the immune system exerts a relentless pressure on surviving tumor cells, ultimately leading to tumor escape from the exhausted immune system. The initial elimination phase is therefore critical to restrict tumor growth very rapidly to prevent relapse or metastasis. Efficient recognition and elimination of transformed cells implies constant monitoring of the body by both the innate and adaptive immune systems, a process called cancer immunosurveillance. Upon monitoring spontaneous, carcinogen- or genetically-induced tumor development in mice bearing various immune deficiencies, critical roles in cancer immunosurveillance have been uncovered for αβ and γδ T cells, NKT cells and NK cells, as well as for the cytokines IFN-γ, IFN-I, IL-12 and for the cytotoxic effector molecules Perforin and TRAIL (50). The role of cDC1 in cancer immunosurveillance remains to be assessed. However, a wealth of data has accumulated on their role in the spontaneous immune rejection of tumor grafts in mice, a popular surrogate model for immunosurveillance (Table 2) (35, 51, 54–58).

**Table 2:** Tumor cell lines spontaneously rejected in immunocompetent hosts.

**Tumor rejection**	**IFN-I-dependent**	**IFN-I-independent**
Lost in *Batf3^−/−^* mice	1969 (51–53) 1773RS100 (35) B16.SIY (54) d38m2 (51) d42m1 (51) GAR4.GR1 (51) H31m1 (35, 51) ***Ptgs1/Ptgs2**^−/−^* **BRAF**^**V600E**^ (55, 56)	
ND	7835 (51) MC-57.SIY (54, 57, 58) P198 (51)	F515 (51)

*Bold: NK cell dependent rejection. Underlined: NK cell independent rejection. Fibrosarcoma: 1969, 1773RS100, 7835, d38m2, d42m1, F515, GAR4.GR1, H31m1, MC-57.SIY. Melanoma: B16.SIY, Ptgs1/Ptgs2^−/−^ BRAF^V600E^. Mastocytoma: P198. 129/SvEv background: 1773RS100, d38m2, d42m1, F515, GAR4.GR1, H31m1. C57BL6/J background: 1969, 7835, B16.SIY, MC-57.SIY, Ptgs1/Ptgs2 ^−/−^ BRAF^V600E^. DBA/2 background: P198*.

## Batf3^−/−^ Mice Fail to Reject Syngeneic Tumor Grafts, Suggesting a Critical Role for cDC1 in Spontaneous Antitumor Immune Defenses

Most tumor cells are not able to directly prime naïve T cells, due to their low expression of MHC class I and co-stimulation molecules or to their acquisition of immunosuppressive functions such as high expression of ligands for immune checkpoint receptors. Thus, induction of CTL responses against most tumors requires accessory cells able to take-up, process and present exogenous tumor Ag in association with MHC-I molecules, a process known as cross-presentation. cDCs are highly efficient in initiating and globally orchestrating adaptive immunity, due to their professional capacities to simultaneously deliver all necessary signals to T cells, namely Ag presentation as signal 1, co-stimulation as signal 2, and cytokines as signal 3 (59). Mouse and human cDC1 excel at activating CTLs, due to their higher capacity to cross-present cellular Ag as compared to other types of Ag-presenting cells (11). It seemed therefore logical that cDC1 should play a critical role in anti-tumor immunity (Table 2). Kenneth Murphy's group was the first to confirm this hypothesis, by showing loss of spontaneous rejection of transplantable tumors in *Batf3*^−/−^ mice (35). Contrary to their WT counterparts, *Batf3*^−/−^ cDCs failed to induce proliferation of OT-I cells when co-cultured with cells loaded with the OVA protein, suggesting that cross-presentation of cellular Ag indeed constitutes one of the critical, non-redundant functions of cDC1. Several other studies have since reported similar results, altogether using a variety of transplantable tumors (Table 2). These studies strongly support a critical role for cDC1 in spontaneous antitumor immune defenses. However, a possible role of the loss of *Batf3* expression in cDC2 or in effector T cells has not been ruled out. In addition, *Batf3*^−/−^ mice can still achieve partial tumor control and mount tumor-specific CTL response under low-dose tumor challenge (35, 51, 54), which might be explained either by the incomplete cDC1 loss or by partial redundancy between cDC1 and other cell types for the cross-presentation of cellular Ag and the induction of antitumor adaptive immunity. Further studies are warranted to address these issues.

## Insights into How cDC1 Could Promote Protective Spontaneous Antitumor Immunity

### Cross-Presentation by cDC1 Is Necessary but Not Sufficient for Immune Control of a Regressor Fibrosarcoma

**Figure 1:**
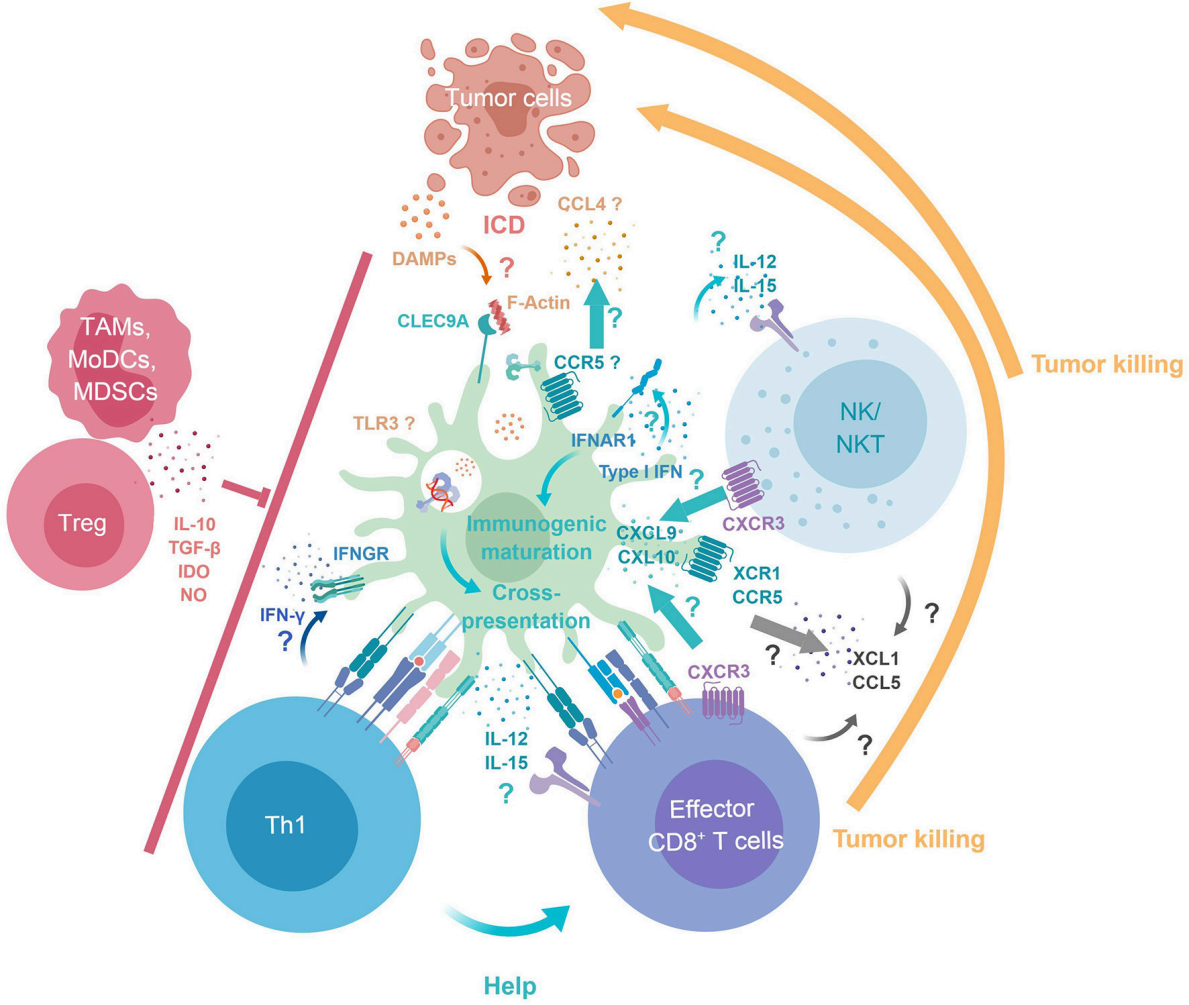
cDC1 key functions in antitumor immunity. Tumor DAMPs and Ag are released upon immunogenic cell death. cDC1 selectively express Clec9A, which binds F-Actin exposed at the surface of necrotic cells, enabling intracellular trafficking of engulfed Ag into endosomes specialized in cross-presentation. cDC1 immunogenic maturation and cross-presentation is promoted by their cell-intrinsic responses to IFN-I. XCR1 and CCR5 expression by cDC1 may contribute to their recruitment by CTL/NK/NKT producing XCL1 and CCL4/5 and by tumor cells producing CCL4. Reciprocally, cDC1 produce CXCL9/10 for local recruitment of CTL/NK/NKT. cDC1 deliver positive co-stimulation and produce IL-12, IFN-β, and IL-15Rα/IL-15 promoting the survival and proper activation of NK and CTL. cDC1 promote Th1 induction and CD4^+^ T cell help delivery to CTLs through simultaneous presentation of Ag in association to MHC-I and MHC-II. CTLs, NK, NKT cells can mediate tumor killing/cell death. Immunosuppressive cells infiltrating the tumor (TAMs, MoDCs, MDSCs, and Tregs) can dampen cDC1, Th1, CTLs, NK, and NKT antitumor immune responses. DAMPs, danger associated molecular patterns; F-Actin, filamentous actin; ICD, immunogenic cell death; MDSCs, Myeloid-derived suppressor cells; MoDCs, Monocyte-derived dendritic cells; TAMs, Tumor associated macrophages; Tregs, Regulatory T cells.

The importance of cross-presentation in cancer immunology has been extensively reviewed (60). Very recently, the WDFY4 molecule, a member of the BEACH (Beige and Chediak-Higashi) domain–containing family of proteins, was reported to be specifically required for cross-presentation of cell-associated Ag by cDC1, and for cDC1-dependant immune control of the highly immunogenic 1969 regressor fibrosarcoma (52). The demonstration of a cell-intrinsic requirement of WDFY4 in cDC1 for immunity against cancer was achieved by comparing tumor growth between *Wdfy4*^−/−^:WT vs. *Wdfy4*^−/−^:*Batf3*^−/−^ mixed bone marrow chimera mice. Importantly, *Wdfy4*^−/−^ cDC1 were not compromised in their abilities to produce IL-12 and to present Ag in association with MHC class II molecules for CD4^+^ T cell activation. *Wdfy4*-deficient cDC1 appeared to be selectively impaired in their ability to cross-present Ag but not in other functions also required for efficient CTL priming and expansion. To our knowledge, this study is the first to demonstrate that a specific defect in cDC1 cross-presentation *in vivo* leads to a failure of mice to control spontaneously tumor growth. Further studies are warranted to confirm these data and extend it to other preclinical tumor models. A major role of *Batf3* in cDC1 is to sustain their expression of *Irf8*. Consistent with this, the development of cDC1 and their ability to cross-present cell-associated Ag are rescued in *Batf3*^−/−^ animals transgenic for *Irf8*. Nevertheless, these mice still fail to control the growth of a regressor fibrosarcoma, likewise to *Batf3*^−/−^ animals. Thus, in addition to cross-presentation, other functions of cDC1 are also necessary for the promotion of protective antitumor immunity but remain to be identified (53). Moreover, both in mice and humans, cDC2 and pDCs can also perform cross-presentation of cell-associated Ag, under specific conditions of stimulation, less efficiently than cDC1 (11). Hence, we propose that cDC1 play a critical role in antitumor immunity not only due to their strong cross-presentation activity but rather because they uniquely combine several key features that are not simultaneously expressed together in other cell types, as detailed below (Figure 1).

### Proposed Key Features Underlying cDC1 Non-redundant Role in Anti-tumor Immunity

First, the expression of XCR1 and CCR5 by cDC1 may enable their local recruitment by cytotoxic lymphocytes producing the ligands for these chemokine receptors, XCL1 and CCL4/5 (45, 55, 61–63). Second, reciprocally, cDC1 ability to produce high levels of CXCL9/10 may promote local recruitment of effector and memory CTLs expressing CXCR3 (43, 57, 64). Third, cDC1 can deliver positive co-stimulation signals. Fourth, cDC1 are a major source of IL-12, IFN-β, and IL-15, thereby promoting the survival and proper activation of NK, NKT cells and CTLs (43, 65–69). In a model of lung metastasis, cDC1 were the major source of IL-12, which was critical to control metastasis in a NK cell- and IFNγ-dependent manner (66). Fifth, cDC1 can promote Th1 induction (70–72) and favor CD4^+^ T cell help delivery to CTL through simultaneous presentation of Ag in association to MHC-I and MHC-II (73, 74). Depending on the cues that they receive during their activation at the time of Ag processing and presentation, DCs will polarize into different functions during their maturation (75, 76). At steady state, during their homeostatic activation, DCs acquire the ability to induce immune tolerance by causing the death, anergy or polarization into regulatory functions of self-reactive T cells, a process referred to as DC tolerogenic maturation. On the contrary, in proper activating contexts, DCs undergo an immunogenic maturation by acquiring the combined expression of activating co-stimulation molecules and cytokines leading to the induction of strong Ag-specific effector lymphocyte responses. The immunogenic maturation of cDC1 is promoted by IFN-I (51, 54, 68, 75), including through cross-talk with pDCs as a major source of these cytokines (77). Cell-intrinsic responses of cDC1 to IFN-I appear to be critical for spontaneous tumor rejection by enhancing their cross-presentation capacity (51, 54), and perhaps also their trans-presentation of IL-15 which promotes the proliferation and effector differentiation of CTLs (68). However, cross-presentation was not totally abolished in *Ifnar1*^−/−^ DCs (51, 54). Upon exposure to high doses of Ag *in vitro*, cross-presentation was even as efficient in *Ifnar1*^−/−^ DCs as in WT DCs. Although, in spontaneously rejected tumor grafts, the cellular source of IFN-I was identified as expressing CD11c, IFN-β production was not altered in *Batf3*^−/−^ mice. Further investigations are required to assess the roles of different types of DCs in CTL activation and in the production of, or responses to, IFN-I, during spontaneous tumor control. In summary, cDC1 constitute a versatile and efficient platform for CTL activation by uniquely bridging several components of innate and adaptive immune responses in a manner promoting mutually beneficial cross-talk (Figure 1). However, further studies are warranted to determine whether the different mechanisms detailed above are each critical for the protective antitumor functions of cDC1, as well as their respective importance.

### When and Where Are cDC1 Functions Exerted During Cancer Immunosurveillance?

Intra-tumoral cDC1 have been suggested to be crucial for *in situ* maintenance of the effector functions of pre-activated CTLs (65). cDC1 promote memory CTL recall upon secondary infections (43). In an experimental model of established immune memory, only tumors that could be infiltrated by both cDC1 and CTLs were spontaneously controlled (57). While conditional cDC1 depletion was not performed in these settings to functionally confirm the importance of cDC1 for the reactivation of antitumor CTLs, this point was addressed in another study examining the reactivation of adoptively transferred antitumor central memory CTLs into WT vs. *Batf3*^−/−^ recipient mice (78). In conclusion, cDC1 might not only be required for the initiation of adaptive immunity against intracellular pathogens or tumors but all along the life cycle of CTLs, including for their maintenance in the tumor as well as for the generation and recall of memory to prevent relapse or metastases.

Several studies have suggested that T cell priming in the tumor draining lymph node is required to mount anti-tumor immunity (79, 80) A study showed that tumor-associated cDC1 bearing intact tumor Ag traffic to the draining lymph node to prime naïve CTLs in a CCR7-dependent manner (80). However, *Ccr7* knock-out had little impact on tumor growth (80). Moreover, CTL priming, activation, proliferation and effector function acquisition in tumor was observed when T cell egress from lymph nodes was blocked (81) or in mice lacking lymph nodes (82). Although these experimental settings could alter cDC1 and lymphoid cell trafficking (83, 84), they nevertheless show that the activation of antitumor adaptive immunity can occur directly at the tumor site (82), possibly in tertiary lymphoid structures developing locally (85). In any case, for efficient tumor rejection without relapse or metastases, systemic immunity is likely important in addition to *in situ* responses, as recently appreciated in the context of immunotherapy (86).

### Proposed Model of cDC1 Role in Antitumor Immunity

**Figure 2:**
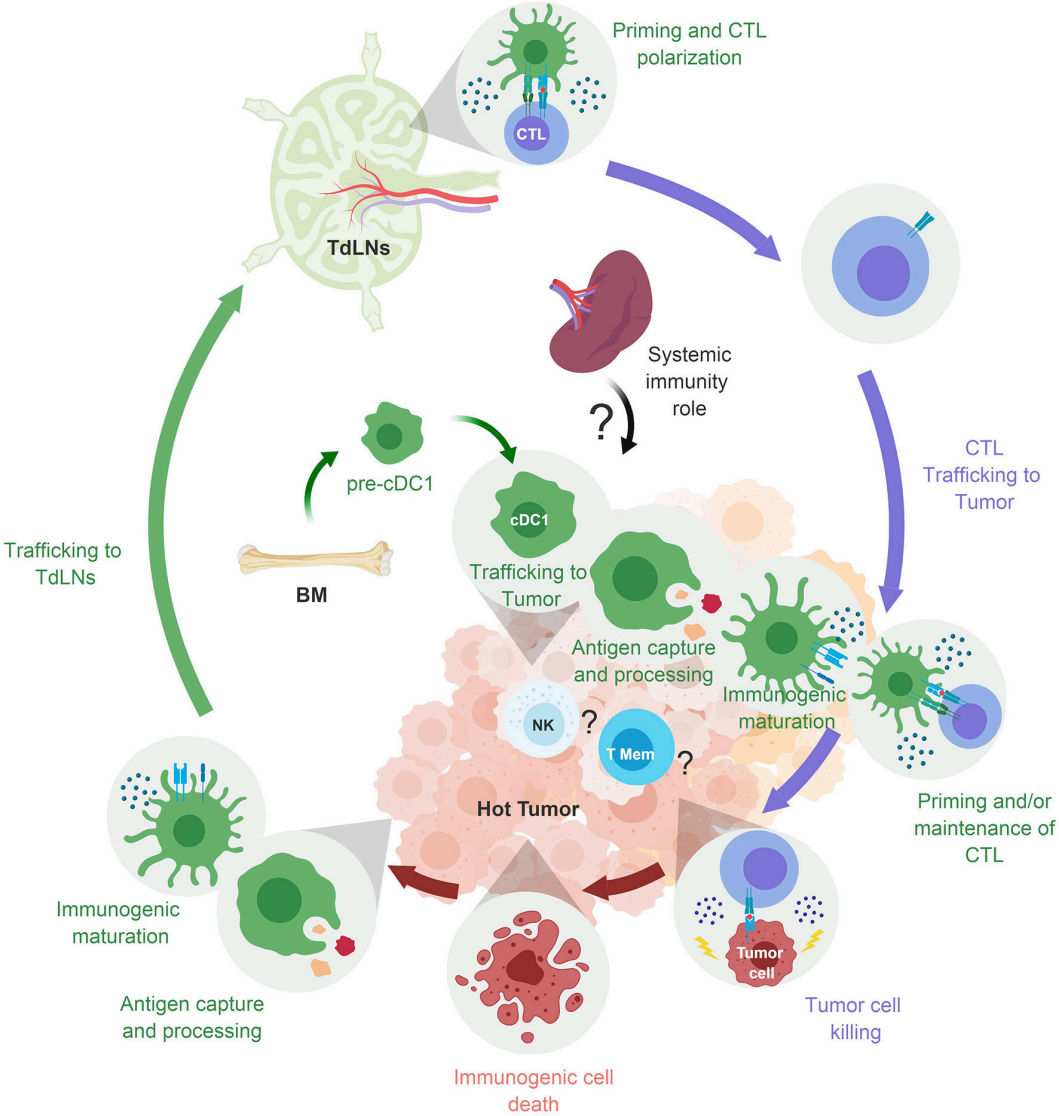
cDC1 cancer immunosurveillance cycle. cDC1 traffic to hot tumor. They uptake cell-associated Ag in the tumor after immunogenic cell death, undergo immunogenic maturation, and traffic to the tumor-draining lymph node. cDC1 prime naïve CTLs and polarize them toward protective effector functions. CTLs expand and migrate to tumor where they can be attracted by chemokines secreted locally by cDC1. The tumor-associated cDC1 also sustain infiltrating CTL protective functions (expansion, maintenance, and memory recall). They might also prime naïve CTLs *in situ*. TdLN, Tumor draining lymph node.

Based on the knowledge discussed in the previous sections, we propose a putative model of the mechanisms through which cDC1 promote the rejection of syngeneic tumor grafts in preclinical mouse models (Table 2) and may physiologically contribute to cancer immunosurveillance (Figure 2). cDC1 take up cell-associated Ag in the tumor after immunogenic cell death, undergo immunogenic maturation, and traffic to the tumor-draining lymph node. There, cDC1 prime naïve CTLs and polarize them toward protective effector functions. CTLs expand and migrate to tumor, where they can be attracted by chemokines secreted locally by cDC1. The tumor-associated cDC1 also sustain infiltrating CTL protective functions (expansion, maintenance and memory recall), and might also prime naïve CTLs *in situ*.

## Failure of immunosurveillance: are cDC1 Direct Targets of Tumor Escape Mechanisms?

We propose a classification of tumors (Hot/Warm/Cold/Icy) according to their immunogenicity, their cDC1 infiltration, maturation and phenotype, and the characteristics of the antitumor CTL response.

“Hot” tumors are characterized as strongly infiltrated by effector CTLs. They are spontaneously controlled by the immune system (Figure 2). They include the syngeneic cancer cell lines used to study spontaneous rejection of tumor grafts (Table 2).

**Figure 3:**
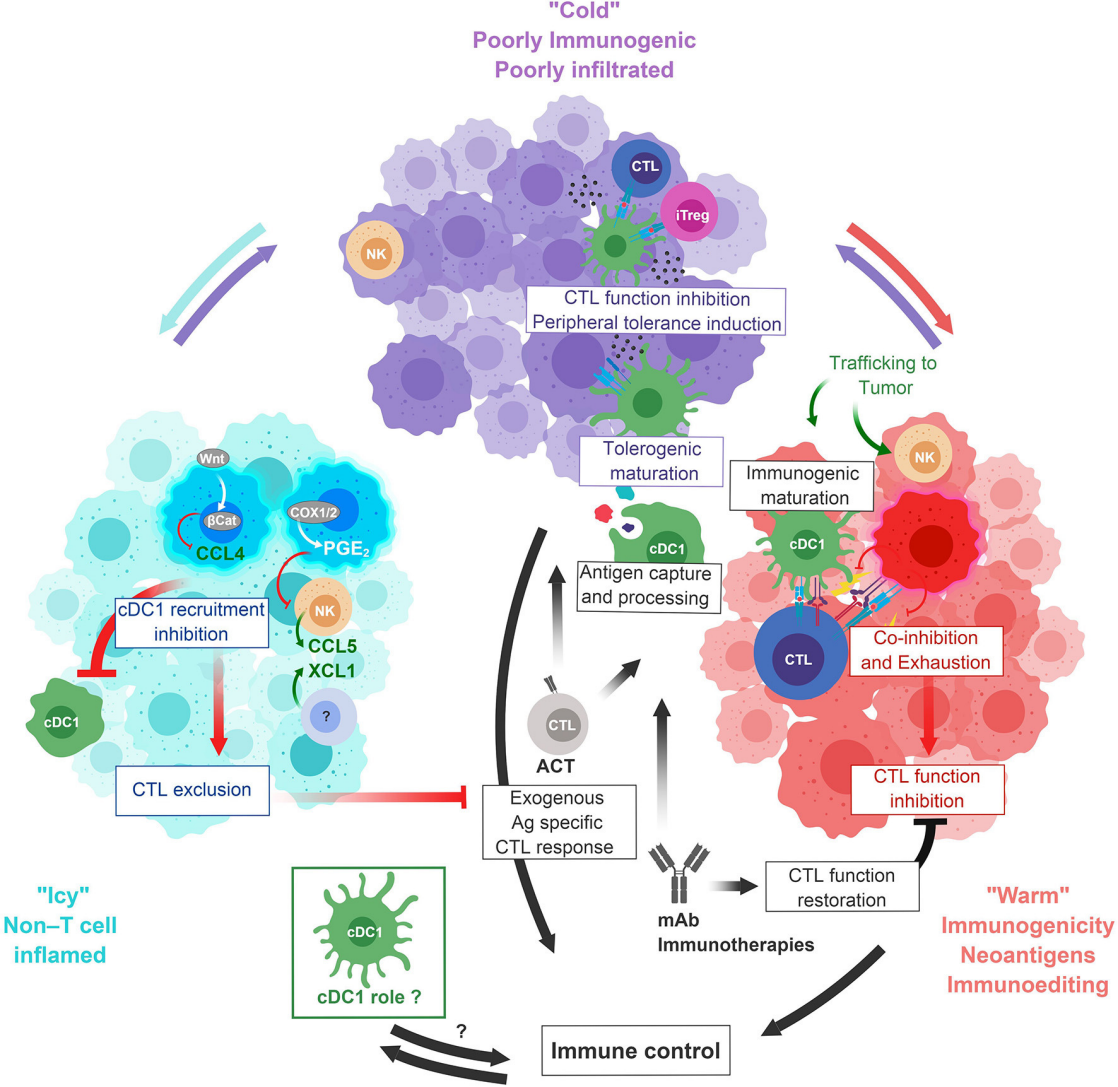
Icy, Cold, and Warm tumors escape from cDC1 immunosurveillance. Icy tumors **(Left)** failed to induce adaptive immune responses. For example, tumors with WNT/β-Catenin signaling or COX elevated activity disrupt the chemokine axes required for local cDC1 recruitment. Impairment of CTL infiltration would occur downstream of the failure of cDC1 to infiltrate the tumor. Cold tumors **(top)** are poorly immunogenic and infiltrated but induce some level of adaptive immunity. Tumors product factors inhibiting cDC1 differentiation or promoting their tolerogenic over immunogenic maturation. This can potentially lead to CTL inhibition and induction of peripheral tolerance. Warm tumors **(Right)** express tumor neoAg and are infiltrated by cDC1 and CTLs but are ultimately not controlled. Cancer immunoediting leads to immune escape. cDC1 have undergone immunogenic maturation but contribute to CTL chronic activation and exhaustion. ACT or mAb immunotherapies could contribute to immune control in Cold and Warm tumors, and cDC1 could play a major role in these settings **(bottom)**. ACT, Adoptive cell transfer; β-Cat, β-Catenin; COX1/2, Cyclo-oxygenase 1/2; iTreg, induced regulatory T cell; mAb, monoclonal antibodies; PGE_2_, Prostaglandin E2.

“Warm” tumors express tumor neoAg and are infiltrated by cDC1 and CTLs (Figure 3 Right). Experimental studies in mice suggest that the correlation between high CTL numbers and increased cDC infiltration in tumors is due to a positive feedback loop between these two cell types mutually promoting their local recruitment and survival. It is not clear how this process is initiated, i.e., which cell type is recruited first to the tumor site. This might depend on the combination of tumor type and host characteristics. “Warm” tumors are ultimately not controlled by the immune system, due to their late selection for harboring immune escape mechanisms, such as intrinsic impairment of Ag processing and presentation (87, 88) or induction of CTL exhaustion (89). In these tumors, cDC1 could have undergone immunogenic maturation but may present Ag to CTL in a manner contributing to their chronic activation and exhaustion, e.g., through engagement of checkpoint receptors such as PD-1 or CTLA4.

“Cold” tumors are weakly immunogenic and poorly infiltrated but induce some level of adaptive immunity (Figure 3 Top). In such tumors, cDC1 could also be direct targets of immune escape mechanisms, such as local production of factors inhibiting DC differentiation or promoting tolerogenic over immunogenic maturation. Those factors include TGF-β, IL-10, IL-6, CSF-1, and VEGF (90). Although cDC1 are proposed to contribute to central and peripheral tolerance (75, 91, 92), whether they can be hijacked by tumors to promote local immunosuppression has not been rigorously investigated.

“Icy” tumors are not immunogenic *per se*, are not infiltrated by T cells and fail to induce immune responses (Figure 3 Left). Those tumors have evaded or hijacked innate immunity in a manner preventing immune cell infiltration at very early stages of the cancer immunoediting process (87). cDC1 can be direct targets of these early immune escape mechanisms. In melanoma, the WNT/β-Catenin signaling pathway prevents the recruitment of cDC1 and CTLs into the tumor, at least in part by inhibiting the local production of CCL4 and CXCL9 (57, 93). CCL4 contributes to the recruitment of cDC1 through their CCR5 chemokine receptor (93). CXCL9 helps promoting the recruitment of both pre-cDC1 (94) and memory/effector T cells (57), through CXCR3. Another mechanism of evasion of innate immunity by melanoma is tumor-intrinsic elevated COX activity leading to PGE_2_ production and downstream inhibition of NK cell, cDC1 and CTL infiltration (55, 56), by disrupting the XCL1/XCR1 and CCL5/CCR5 chemotactic axes. Impairment of CTL infiltration into the tumor is proposed to occur downstream of the failure of cDC1 recruitment (55, 56).

In brief, cDC1 are direct targets of tumor escape mechanisms since the tumor microenvironment can modulate all of the processes necessary to promote their protective antitumor functions. It can determine the tolerogenic vs. immunogenic nature of tumor cell death (95–97), control the expression of the growth factors and chemokines promoting local recruitment, differentiation, expansion and survival of cDC1 or their progenitors (55, 93, 98), dampen cDC1 production of activating cytokines (56, 67), and inhibit their maturation or even polarize it toward tolerance (99, 100).

## Studies of the Natural Role of cDC1 in Immunotherapies

In the last two decades, cancer treatments have successfully shifted from only targeting the cancer itself to also manipulating the immune system, with the aim to boost or induce *de novo* protective antitumor cellular immune responses, mainly CTLs but also NK and NKT cells. These novel treatments called immunotherapies encompass different strategies. Here we will specifically discuss studies performed in experimental settings mirroring the two types of immunotherapies that have shown the best clinical benefits in cancer patients. First, we will focus on treatments providing exogenous effector cells through ACT of autologous antitumor CTLs, after their expansion and activation *in vitro* (eventually combined with genetic engineering for CAR T cells), i.e., CTL ACT. Second, we will discuss mAb immunomodulation (mAIM) to block checkpoint receptors on CTLs or NK cells (101–106), i.e., ICB, which has proven more efficient than conventional chemotherapies or radiotherapies in several cancer types, with better overall responses, and, most strikingly, significantly increased long-term survival (107). ACT or ICB monotherapy promotes durable disease control only in 30% of the patients. While they dramatically improve the response rate in patients with metastatic melanoma, ICB bi-therapies cause significant adverse effects and toxicities, with high incidences of autoimmune manifestations (108, 109). Understanding the mechanisms controlling responsiveness to ACT or ICB is thus a prerequisite before complementing these immunotherapies by adjuvant treatments able to further improve the rate and duration of remission for cancer patients. One hypothesis to explain patient non-response to immunotherapies is an impairment of the accessory cells needed to promote CTL reactivation and to sustain their effector functions, rather than cell-intrinsic defects in the CTLs themselves. In this scenario, cDC1 are likely candidates, based on their critical role in promoting the spontaneous rejection of tumors in preclinical mouse models, and on their unique functional features endowing them with a high efficiency for nurturing cytotoxic cells all along their life cycle.

### Role of cDC1 in Promoting CTL ACT Efficacy

The ACT procedure the most commonly used so far consists in isolating endogenous CTLs from a cancer patient, expanding them *in vitro* through tumor Ag-specific re-stimulation under conditions allowing reversal of exhaustion, and then re-infusing them into the host. By using autologous cells for the treatment, this strategy alleviates any side effects that could arise in allogenic settings. However, one major issue is that only few cancer types respond to this treatment. This might be due to immune escape mechanisms in the tumor limiting locally CTL access to the activating signals necessary to prevent their exhaustion and promote their proliferation, sustained activation and survival. Preclinical mouse models have been used to address this issue, aiming at determining whether professional Ag cross-presentation in the context of positive co-stimulation and delivery of specific cytokines is necessary for ACT efficacy. Since cDC1 excel at this combination of functions (Figure 1), they could promote ACT efficacy. Indeed, injection of diphtheria toxin in ACT recipient *Zbtb46-DTR* mice significantly decreased their response to immunotherapy. cDC1 but not cDC2 from tumor-engrafted control mice were shown to cross-present tumor Ag and produce IL-12 *ex vivo*. Thus, it was concluded that cDC1 are necessary for ACT efficacy in these experimental settings (65). However, opposite results were recently reported under similar experimental conditions, showing a lack of cDC requirement for ACT success (110). Differences between the experimental set-up of these two studies might explain their different conclusions, since only the second study used bone marrow chimera mice rather than directly Zbtb46-DTR animals, which is necessary to rule out any impact of loss of Zbtb46 expression in other cells than cDCs (33). Therefore, additional studies are necessary to determine whether cDCs are required for maximal ACT efficacy, and how. If those studies unravel specific pathways that can be potentiated, this could allow designing of a “DC adjuvant” therapy for ACT, which might broaden its success rate to more patients and for additional cancer types.

### Role of cDC1 in Promoting Responses to mAIM

In the course of a normal immune response, Ag-presenting cells regulate their expression of ligands for T cell activating vs. inhibitory co-receptors. This contributes to fine tune the intensity and kinetics of the adaptive immune response, in order to balance efficient immune control of pathogens with the risk of developing an immunopathology due to an excessive T cell activation. Tumors can hijack this process by expressing ligands for T cell inhibitory receptors leading to premature termination/exhaustion of CTL responses (48, 107). This tumoral immune evasion strategy can be overcome by mAIM through infusion of mAbs capable of either inhibiting the engagement of T cell inhibitory co-receptors (i.e., ICB mAbs) or mimicking the engagement of T cell activating co-receptors (co-stimulation activating mAbs) [listed in (106)]. These mAbs can be used as monotherapy or bi-therapy. The ICB mAbs the most commonly used in clinics are directed against programmed cell death protein 1 (PD-1) and cytotoxic T-lymphocyte associated protein 4 (CTLA-4). Although their use has dramatically improved patient survival for different types of cancer, their precise mode of action is still a matter of debate. The mechanisms underlying lack of response in the majority of patients remain elusive. Preclinical mouse models have been used to address this issue and showed that treatment efficacy is abrogated in cDC1-deficient *Batf3*^−/−^ animals (Table 3) (56, 57, 65, 69, 117, 118). This is the case for anti-CTLA4 (117) or anti-PD-L1 (79) monotherapies, for a bi-therapy combining anti-PD-1 and CTLA4 mAbs (69), and for a bi-therapy combining the anti-PD-1 mAb with the co-stimulation activating anti-CD137 mAb (118). However, these studies did not determine whether the lack of mAIM-dependent tumor control in *Batf3*^−/−^ mice was due to a lack of antitumor CTL priming at the time of tumor engraftment, before immunotherapy, or to a failure of mAIM at inducing the reactivation of previously primed but exhausted antitumor CTLs, at the time when the immunotherapy was administered. Efficient activation of anti-tumor CTLs, for proliferation and acquisition of effector functions, requires cross-presentation of tumor-associated Ag, activating co-stimulation and delivery of specific cytokines from accessory cells. As described previously, cDC1 excel at simultaneously delivering all these signals (Figure 1). In particular, one of the critical functions of cDC1 during mAIM immunotherapies may be to deliver IL-12 (69). In addition, cDC1 may also be a major source of CXCL9/10 (43) for recruiting activated or memory CTLs into the tumors (57, 93) (Figures 1, 2).

**Table 3:** Studies in mouse preclinical cancer models investigating the impact of cDC1 depletion on the protective effects of various immunotherapies not designed to directly target these cells.

**Model of cDC1 depletion**	**Tumor models**	**Therapy used**	**Outcome of cDC1 depletion**	**Remarks**	**References**
CD11c-hDTR *Batf3^−/−^*	B16gp100 (Melanoma)	Drug: Ad.*flagr170* MOA: Immune chaperone inducing NF-κB activity	Beneficial effect lost upon DT injection and in *Batf3^−/−^* mice	DT injection resulted in only a partial loss of protection	(111)
*Batf3^−/−^* CD11c-hDTR	B16-EGFR-SIY (Melanoma)	Drug: anti-EGFR-IFNβ MOA: IFN-I delivery to mAb resistant tumor enhance immune response	Beneficial effect lost upon DT injection but not in *Batf3^−/−^* mice		(112)
*Batf3^−/−^*	Braf^*V*600*E*^ (Melanoma)	Drug: Aspirin/anti-PD-1 MOA: COX inhibition allows a more efficient mAIM therapy	Beneficial effect lost in *Batf3^−/−^* mice	CXCL9/10 source to be further investigated	(56)
*Batf3^−/−^*	B16 (Melanoma) 4T1.2-Neu (Breast Cancer)	Drug: DC-targeted XBP1 plasmid/Tumor Ag MOA: XBP1 enhances DC cross-presenting capacity	Beneficial effect lost in *Batf3^−/−^* mice		(113)
*Batf3^−/−^*	B16F10 (Melanoma)	Drug: IFN-α/TA99+FcIL-2 MOA: IFN-α given after Ag uptake by cDC1 promotes their immunogenic maturation	Beneficial effect lost in *Batf3^−/−^* mice		(114)
*Batf3^−/−^*	B16 (Melanoma)	Drug: BRAFI/anti-PD-L1/poly(I:C) /Flt3-L MOA: Sequentially administered combination therapy targeting DC, CTL and the tumor itself	Beneficial effect lost in *Batf3^−/−^* mice	*Batf3^−/−^*	(79)
*Batf3^−/−^*	BP-SIY (Melanoma) BPC-SIY (Melanoma) MC-57.SIY (Fibrosarcoma)	Drug: Flt3-L-BM-DC/anti-CTLA-4/anti-PD-L1 MOA: Intratumoral injection of BM-DCs induce CTL recruitment	Beneficial effect lost in *Batf3^−/−^* mice	CXCL9/10 source to be further investigated	(57)
*Batf3^−/−^*	B16-mCD20+ (Melanoma)	Drug: anti-Clec9A-IFNα2/TNF/Doxorubicin/anti-PD-L1/anti-CTLA-4/anti-OX40 MOA: IFN-I targeting to cDC1 through Clec9A mAb	Beneficial effect lost in *Batf3^−/−^* mice		(115)
Clec9a-hDTR *Batf3^−/−^* CD11c-hDTR	4T1 (Breast Cancer) B16F10 (Melanoma) B16-OVA (Melanoma)	Drug: poly(I:C) /MSU+M.*smeg* MOA: MSU+M*smeg* and poly(I:C) stimulate cDC1 immunogenic maturation through TLR3. poly(I:C) promotes MoDC recruitment	Poly(I:C) but no MSU+M.*smeg* beneficial effect preserved upon DT injection in *Batf3^−/−^* mice		(116)
*Batf3^−/−^*	d42m1-T3 (Fibrosarcoma)	Drug : anti-CTLA-4 MOA : Injection of anti-CTLA-4 allowing tumor rejection	Beneficial effect lost in *Batf3^−/−^* mice		(117)
*Batf3^−/−^*	B16F10 (Melanoma)	Drug: anti-PD-1/anti-CD137 MOA: Immunostimulatory mAbs used in combination enhance antitumor immunity	Beneficial effect lost in *Batf3^−/−^* mice	IL-12 source to be further investigated	(118)
	MC-38 (Colon Carcinoma)				
*Batf3^−/−^*	AT-3ovadim CD73+ (Breast Carcinoma)	Drug: anti-PD-1/anti-CTLA-4 MOA: Reactivation of endogenous CTLs and depletion of Treg	Beneficial effect lost in *Batf3^−/−^, Il12p35^−/−^* and *Il12p40^−/−^* mice	IL-12 source to be further investigated	(69)
Zbtb46-hDTR	EG-7 (Thymoma)	Drug: *in vitro* pre-activated Ag specific CTL MOA: Ag-specific CTL infiltrate the tumor and delay its growth	Beneficial effect lost upon DT injection	These two studies drew diverging conclusion on cDC1 implication. Further studies are required.	(65)
Zbtb46-hDTR	B16-OVA (Melanoma)	Drug: *in vivo* pre-activated Ag specific CTL MOA: Ag-specific CTL delay the tumor growth	Beneficial effect preserved upon DT injection		(110)

*MOA : Mode of action*.

### Current Limitations, Controversies or Unknowns

Many of the conclusions drawn above are based on the use of *Batf3*^−/−^ mice, or on the assumption that cDC1 are the main source of the cytokines or chemokines promoting response to mAIM therapies, without formal demonstration of this point by functional inactivation of candidate functions selectively in cDC1. Moreover, the respective importance of Ag cross-presentation vs. delivery of specific activating co-stimulation or cytokine signals by cDC1 has not been delineated yet under immunotherapies condition. Thus, further studies are required to confirm and extend these analyses, by using other mutant mouse models allowing specific cDC1 depletion or selective manipulation of each of their candidate functions.

## Harnessing cDC1 Functions to Improve Immunotherapies Against Cancer

In parallel of developing immunotherapies to directly boost lymphocyte effector responses against tumor cells, the community has put much effort in trying to elaborate vaccines to ignite or reactivate endogenous antitumor immune responses in patients. Among all Ag presenting cells identified so far, cDC1 are the only ones to express selectively unique cell surface markers, such as CLEC9A or XCR1, which enables their specific targeting with mAb *in vivo*. Intratumor injection of bone marrow-derived DCs highly enriched in cDC1 increased local CTL infiltration and improved response to ICB (57). Therefore, in combination with other immunotherapies, cDC1 represent a very good candidate immune cell type to mobilize with off-the-shelf compounds for boosting patient antitumor immunity.

### Specific Targeting of cDC1 for Vaccination Purposes

Many preclinical studies in various mouse models have demonstrated the efficacy of *in vivo* targeting of Ag specifically to cDC1 in combination with the administration of a proper adjuvant for priming or reactivating adaptive immunity, leading to a rapid yet long term immune protection against infections by intracellular pathogens or against tumors (119) (Table S1). Adjuvants used to induce a beneficial inflammation promoting an immunogenic environment to prevent or counterbalance tumor immunosuppressive functions include the Toll-Like-Receptor ligands LPS, Imiquimod, CpG or Poly(I:C). Other adjuvants include drugs which directly stimulate accessory lymphocytes, such as αGalCer for NKT cell activation (120, 121), or agonistic anti-CD40 antibodies which mimic the helper signal delivered by CD4^+^ T cells to DCs for promoting their production of the lymphocyte activating cytokines IL-12 and IL-15/IL-15Rα (68, 122, 123). Vaccine formulation including naked DNA (124, 125), porous polymer matrices (126), or oil in water nano-emulsion (127) are intrinsically immunogenic. Vaccination based on macroporous polymer matrices encapsulating tumor lysates, GM-CSF and CpG, were quite effective in attenuating tumor growth (126), although not targeting specifically cDC1. DEC-205 has been by far the cell surface marker the most used to target cDC1 *in vivo* (Table S1). However, it is not specific of cDC1 since it is expressed on Langerhans cells in the epidermis, on all migratory DC in lymph nodes (128) and it is highly upregulated on various DC subsets in tumors (30). The same issue applies to CD40. This raises the question of the respective roles of cDC1 vs. other types of DC in the protection conferred by vaccines based on *in vivo* Ag delivery through DEC-205 or CD40. Indeed, tumor Ag delivery to pDCs or cDC2 by using anti-BST2 (129) or anti-DCIR2 mAb (130) respectively, or administration of tumor Ag-pulsed pDC (131), are highly efficient in conferring protection against cancer. This shows that not only cDC1 but also cDC2 or pDCs can induce protective antitumor immunity, providing that Ag is delivered to these cells through adequate endocytic receptors in the presence of proper maturation signals. Interestingly, immunization with tumor-associated exogenous cDC1 or cDC2 prior to tumor engraftment revealed complementary functions of these two DC types (30). In a model of challenge with Lewis Lung carcinoma, only cDC2 vaccination led to reduced tumor growth rate and weight, correlating with reduced tumor infiltration by myeloid-derived suppressor cells, functional polarization of tumor-associated macrophages toward a M1-like antitumor phenotype, and promotion of Th17 rather than Treg CD4^+^ T cell responses (30). cDC1 were confirmed to be more efficient than cDC2 for the induction of antitumor CTL responses, which protected against a challenge with B16 melanoma (30).

In summary, even though other DC types can be successfully harnessed for cancer vaccines in mouse preclinical models, many studies showed that *in vivo* targeting of cDC1 is highly efficient for the activation of antitumor CTL responses able to induce complete tumor rejection in prophylactic settings and to delay significantly tumor progression or metastasis in therapeutic settings (Table S1). The efficacy of DC-targeted vaccines depends on three critical parameters: (i) the mode of delivery of the Ag, (ii) the nature of the Ag, and (iii) the nature of the adjuvant. Targeting Ag to cell surface receptors trafficking into late endosomes or lysosomes promotes more efficient cross-presentation by human cDC1 as compared to cDC2, whereas both cell types can mediate this function upon Ag delivery to early endosomes (132). The route of administration of the vaccine should be carefully determined depending on the necessity to induce mucosal and/or systemic immunity according to the type of cancer involved (86, 133). Once activated, tumor-associated DCs have the capacity to migrate to tumor-draining lymph nodes to prime T cells (80) and may rather favor a local antitumor immunity. There is also evidence that CTL priming can occur directly in the tumor (82). Hence, intra- or peri-tumoral administration of cDC1-targeted Ag for solid tumors may be the best way to enhance priming of CTLs both inside the tumor, and through migration of tumor-associated DCs to the draining lymph node. The tumor Ag should be well selected as the immune system can be almost irreversibly tolerized against certain self Ag (134). Some adjuvants are more efficient in promoting a beneficial inflammatory microenvironment in the tumor, linked to their ability to induce IFN-I. It might be desirable to include adjuvants that directly engage cDC1 since exposure to inflammatory mediators in the absence of direct signaling by pattern recognition receptors might not be sufficient to promote immunogenic DC maturation (135).

### Mobilizing cDC1 Functions in Combination Immunotherapies

In most of the preclinical models discussed above, cDC1-targeting therapeutic vaccines delay tumor progression or metastasis, or even promote a better tumor control over a long time, but fail by themselves in inducing complete tumor rejection. However, combining strategies mobilizing cDC1 with current immunotherapies, in particular with ICB, should promote the induction of long lasting protective antitumor immunity in more patients, and should more generally improve the objective response rate, the response duration and the overall survival of patients. In preclinical mouse models of immunotherapies, the antitumor effects of various off-the-shelf treatments were shown to require cDC1 functions (Table S1), and a variety of strategies were specifically designed to harness cDC1 against cancer (Table 4). Hereafter, we discuss how these studies advanced our understanding of when, where and how to mobilize cDC1 functions in combination immunotherapies.

**Table 4:** Anti-tumor off-the shelf therapies relying on cDC1 functions.

**Mode of action**		**Therapy**	**Putative “cDC1 booster” and protocol of administration**	**Type of tumors**	**Depend on**	**Does not depend on**	**Effects on cells**	**Impact of cDC1 depletion**	**References**
Promoting maturation		Cytokine therapy (Fc-IL-2)	IFN-I delivery, 48h after induction of ADDC against tumor upon administration of an anti-tumor mAb	B16-F10 DD-Her2/neu breast cancer RM9 prostate cancer	CD8α+ cells CSF1R+ cells IFN-γ	IFN-γ-production by CTL is Batf3-independent	↑ CTL activation ↑ cDC1 maturation and tumor uptake Early influx of neutrophils ↑ production of chemokines	Delayed control in *Batf3^−/−^* mice	(114)
Blocking checkpoint inhibitors on cDC1 (and putatively on other cells)		Chemotherapy	anti-TIM3 antagonist mAb before chemotherapy	PPMTV-mCherry	IFN-I IL-12 IFN-γ CXCR3 CD8α+ cells	n.d.(*)	Potentiates CTL activation No effect on IL-12 production by cDC1 ↑ chemokine secretion by cDC	↑ growth in *Batf3^−/−^* and *Itgax-Cre; Irf8*^fl/fl^ mice or in Zbtb46-hDTR BM chimera	(64)
		Chemotherapy	anti-galectin9 mAb before chemotherapy (Gal9 = Tim3 ligand)	PPMTV-mCherry	CXCR3 CD8β+ T cells	n.d.	n.d.	n.d.	(64)
Providing cytokine support for T cell reactivation and polarization		mAIM (Anti-CD137)	Recombinant IL-12 after mAIM (mimicking boosting of IL-12 production by cDC1)	MC38 s.c	n.d.	n.d.	↑ tumor control	no effect of IL-12 in *Batf3^−/−^* mice	(118)
Expansion of cDC	Promoting cytokine-production	mAIM (anti-PD-L1)	FLT3-L (for 9 consecutive days) + 2 inj. Poly(I:C)	B16 BRAF^V600E^; PTEN melanoma	n.d.	n.d.	↑ CTL activation and infiltration in tumor	n.d.	(79)
		mAIM (anti-PD-1 or anti-CD137)	Hydrodynamically injected iv on the day of engraftment 1 inject. Poly(IC:LC) i.t. 7d after	B16-OVA s.c.	n.d.	n.d.	n.d.	↑ growth in *Batf3^−/−^* mice	(118)
		Radiation	Poly(I:C) 1 d before radiation	LLC-OVA s.c. (BALB/c)	CD8β+ T cells TNF (by improving ionizing radiation effect)	n.d.	↑ CTL activation in LN, spleen and tumor ↑ CXCL10 and IFN-β	↑ growth in *Batf3^−/−^* mice	(136)
		mAIM (anti-PD-L1 + anti-CTLA-4)	FLT3-L 1/week for 4 weeks	RCC Xenograft: Renca cells s.c. (BALB/c)	n.d.	n.d.	↑ CD103^+^ cell and CTL tumor infiltration↑ CTL activation	n.d.	(137)

*All tumor models were engrafted in C57BL/6J unless otherwise specified. LCC, Lewis Lung Carcinoma; RCC, Renal Cell Carcinoma; Fc, fragment, crystallizable region of murine immunoglobulin G2a (IgG2a); ↑increased; n.d., not determined*.

* *Of note: anti-TIM3 alone exerts its effect independently of CD11c+ cells (138)*.

Upon immunotherapy, cDC1 are increased in the tumor very early after the beginning of the treatment, and have left the tumor in favor of cDC2 during the phase of immunotherapy-induced rejection of the tumor (86). Therefore, the location and timing of cDC1 booster administration in combination with immunotherapies are likely to be determinant for treatment success.

One way to attempt improving the response of cancer patients to immunotherapy is to boost the ability of their cDC1 to cross-present tumor Ag (60). Combined administration in mice of mAbs directed against tumor Ag with a stabilized form of IL-2 enhances antitumor immunity in a cDC1-dependent manner (Table 4) (114, 139). This is because antibody-dependent cell-mediated cytotoxicity provokes an immunogenic tumor cell death favoring the up-take and cross-presentation by cDC1 of tumor cell fragments. Indeed, tumor cell lysates or tumor plasma membrane vesicles may represent the best sources of Ag for cross-presentation, because they include a constellation of neoAg. Tumor Ag cross-presentation by cDC1 can also be triggered upon administration of tumor Ag coupled to mAb directed against cDC1 surface markers (Table S1).

Cross-presentation of tumor Ag by cDC1 must occur simultaneously to their immunogenic maturation such that they can deliver all of the signals required for the efficient priming of naïve CTLs or the reactivation of exhausted CTLs, including proper co-stimulation, activating cytokines, chemokines and CD4^+^ T cell help, in the tumor bed or upon migration to the draining lymph node. This implies administrating the good adjuvant at the right time and in the proper place. TLR3, CpG, or STING agonist adjuvants promoting a strong production of IFN-I are especially efficient at promoting antitumor immunity, even more upon peritumoral rather than systemic delivery (79, 140–142). To further promote the beneficial anti-tumor activity of IFN-I and limit their deleterious side effects, a synthetic mutated IFNα2 has been engineered and coupled to anti-Clec9a mAb, allowing delivery of IFN-I activity specifically on cDC1. The administration of this cDC1-targeted adjuvant synergizes with mAIM, chemotherapy, or with low dose of TNF, resulting in a regression or a long-lasting protection against melanoma and breast carcinoma in the absence of toxic effects (115). Targeting IFN-I on tumor cells also improves the antitumor effects of mAIM (112, 114, 143), in part through direct effects on cDC1 and/or cDC2 (143) but also more generally by modulating the responses of many other immune cells in the tumor microenvironment. Importantly, to promote protective antitumor immunity, IFN-I must be delivered simultaneously to, or shortly after, the tumor Ag. Indeed, IFN-I-induced cDC1 maturation strongly decreases their phagocytic capacity and thus prevents their ability to cross-present if occurring before tumor Ag uptake (114).

IL-12 production by cDC1 is proposed to significantly contribute to their protective antitumor activity, at least in part by promoting Th1 response and activating IFN-γ production by NK cells and CTLs. Administration of recombinant IL-12 in combination or not with mAIM therapy displayed anti-metastatic (66) or immunotherapy-induced antitumor effect (118) in WT animals (Table 4). However, interestingly, these potentiating effect of the mAIM therapy was lost in *Batf3*^−/−^ mice (118), showing that IL-12 administration is not sufficient to replace the antitumor functions of cDC1.

Another function of cDC1 that could be exploited for boosting current immunotherapies is their ability to respond to the chemoattractant XCL1, due to their specific expression of the chemokine receptor XCR1. At steady state, high levels of the *Xcl1* transcript are detected in NK cells, NKT cells and memory CTLs. Upon activation, *Xcl1* expression is further upregulated in these cells and induced in effector CTLs, which promotes the recruitment of cDC1 into inflamed tissues in close contact to XCL1-producing cells, leading to a cross-talk amplifying the responses of both cell types (77). Therefore, intra-tumoral delivery of XCL1 seemed a promising strategy to enhance local recruitment of cDC1 in order to harness their protective functions in combination immunotherapies. Certain types of melanoma or colon carcinoma tumors engineered to express high amount of XCL1 harbored a higher cDC1 infiltration and were rejected faster or grew more slowly in WT but not in *Batf3*^−/−^ mice, as compared to control tumors. However, this process was inhibited in tumors producing PGE2, due in part to the ability of this molecule to decrease XCR1 expression in cDC1 (55). This study illustrates well the necessity not only to mobilize cDC1 in combination immunotherapies, but at the same time to dampen the immunosuppressive pathways targeting cDC1 functions in the tumor microenvironment. Hence, in addition to directly targeting CTL and cDC1 functions, combined immunotherapies should probably include means to counteract the tumor immunosuppressive pathways acting indirectly on these cells, such as inhibiting β-catenin, PGE_2_ or adenosine receptor signaling (55, 57, 93, 144), or depleting/reprogramming the tumor-associated mononuclear phagocytes endowed with immunosuppressive functions including macrophages, MDSCs and pDCs (67, 145, 146).

Because cDC1 are the rarest subset of Ag presenting cells in tumors (30) and their numbers have been shown to decrease in the course of certain immunotherapies (86), strategies aiming at harnessing their functions for cancer treatment should include methods to promote their expansion *in vivo*. Tumor-infiltrating NK and T cells upregulate FLT3-L, which seems to contribute to the local expansion of tumor cDC1 (62), and most likely cDC2. Administration of recombinant FLT3-L to tumor-bearing mice as a supportive treatment to mAIM immunotherapy reinforces CTL infiltration and activation in the tumor (137), and the combined administration of FLT3-L and poly(I:C) which respectively support cDC1 expansion and activation significantly improved antitumor mAIM immunotherapy in mice (79, 118) (Table 4). Alternatively, large quantities of cDC1 could be injected peritumorally simultaneously to ICB administration, in order to further promote the priming of naïve CTLs toward neoAg or the reactivation of endogenous antitumor CTL responses. This should be achievable since recent studies showed that large numbers of fully functional cDC1 can be generated *in vitro* from hematopoietic progenitors cultured with FLT3-L on feeder cells expressing the Notch ligand Delta-like 1 (147, 148).

In summary, several studies have attempted to improve the response to cancer chemotherapies, radiotherapies or mAIM immunotherapies by combining these treatments with putative or known cDC1 boosters (Table S1 and Table 4). In all cases, tumor progression was greatly dampened in parallel with enhanced CTL activation and sometimes with a documented increased maturation of cDC1. In many studies, this beneficial effect was shown to be abrogated in *Batf3*^−/−^ mice. These studies in mouse preclinical models of combined immunotherapies strongly enforce the hypothesis that harnessing cDC1 functions in cancer patients should improve their response rate and long-term survival to already existing immunotherapies including ICB, and show how this could be achieved.

## What Functional Specificities Make Human cDC1 Good Candidate Ag-presenting Cells for the Promotion of Protective Anti-tumor Immunity?

### Comparative Genomics Established Overall Homology Between Mouse and Human cDC1

A striking overall homology between human and mouse cDC1 was established through cross-species comparative genomics of several immune cell types (14, 149–153). This provided a very strong incentive to investigate the role of human cDC1 in antitumor immunity, considering the body of evidence discussed above supporting a critical role of mouse cDC1 in promoting NK- and CTL-mediated tumor control in preclinical cancer models.

### Conservation of Key Characteristics Proposed to Underlie Mouse cDC1 Protective Role Against Cancer

A number of shared and distinctive features of mouse and human cDC1 are summarized in Table 5 (11, 14, 15, 61, 63, 79, 147–150, 154–162, 165–174), with their possible relevance for immune defense against cancer. Globally, the combination of features proposed to endow mouse cDC1 with their unique efficacy to promote protective anti-tumor immunity is well conserved in human cDC1. Differences in cross-presentation efficacy appear to be more subtle between human than mouse DC subsets (155, 175). Of note, however, a consensus has emerged from various studies that human cDC1 are more efficient than other DC types for the cross-presentation of cell-associated Ag (15, 45, 63, 155–157), likewise to the situation in the mouse. Human cDC1 were reported by several teams not to produce IL-12 (150, 167). However, other studies have shown that under optimal conditions of stimulation human cDC1 can produce this cytokine to levels equivalent or higher than those made by cDC2 or MoDCs (147, 156, 168, 169, 173, 174).

**Table 5:** Shared and distinctive features of mouse and human cDC1.

**Feature**	**Present in mouse cDC1**	**Present in human cDC1**	**Relevance to anti-tumor immunity**	**References**
Dependency on IRF8 and NOTCH signaling for differentiation	YES	YES	Not applicable	(147, 148, 154)
High efficiency for cellular Ag cross-presentation	YES	YES	Cross-presentation of tumor-associated Ag	(15, 45, 63, 155–157)
Expression of CLEC9A	YES, shared with pDCs	YES	Intracellular routing of engulfed tumor Ag in endosomes specialized in cross-presentation	(158–160)
Higher efficacy for cytosolic export of engulfed proteins	YES, specific to cDC1	YES, shared with other DC types	Cross-presentation of tumor Ag	(155, 161)
Alkaline endosomes	YES, specific to cDC1	YES, shared with cDC2	Limits the degradation of endocytosed tumor Ag to favor their cross-presentation	(155, 162)
Selective high expression of *RAB11A, RAB7B, RAB43* and *SEPT3*	YES	YES	Small RAB GTPases with documented or putative role in promoting Ag cross-presentation	(14, 163–165)
*GCSAM* (*GCET2*), *CLNK, SNX22* and *WDFY4* expression	YES, Clnk expression shared with NK and mast cells	YES, CLNK expression specific to cDC1	WDFY4 involved in cross-presentation; other gene functions in cDC1 unknown	(11, 14, 52, 165)
CADM1 (IGSF4A) expression	YES	YES	CTL activation?	(166)
Specific expression of XCR1	YES	YES	Local recruitment of cDC1 by, or stabilization of their interactions with, NK cells and CTLs	(14, 45, 61, 63)
High TLR3 expression and specific production of IFN-βand IFN-λs upon TLR3 triggering	YES IL-12 induced as well	YES, high IL-12 production observed in some but not all studies	•Putative source of IFN-β/λs in tumors, promoting DC maturation and CTL activation? •Therapeutic target to promote immunogenic inflammation in combined immunotherapies	(15, 79, 147, 150, 156, 167–170)
TLR9 and TLR11 expression and production of IL-12 upon their triggering	YES, shared with other DC subsets for TLR9	NO, TLR9 not expressed in human cDC1, no TLR11 ortholog in humans	Not applicable in humans	(171, 172)
TLR8 expression and production of IL-12 upon its triggering	NO, loss of TLR8 ligand binding in mice	YES, under adequate conditions of stimulation	•Local recruitment and activation of CTL and NK cells •Therapeutic target to promote immunogenic inflammation in combined immunotherapies	(147, 173, 174)

### Current Limitations, Controversies or Unknowns

One study has recently reported that human cDC1 do not migrate efficiently from the parenchyma of non-lymphoid tissues to their draining lymph nodes (176). This bears important implications for vaccination or immunotherapies if it is confirmed.

The mechanisms that make human cDC1 especially efficient for cross-presentation of cell-associated Ag are still not understood. One of the main limitations to address this issue, and more generally to study the functions of human cDC1 and their molecular regulation, is their rarity and fragility.

## What Evidences Exist that Human cDC1 Correlate with a Better Outcome in Cancer Patients and what can be Inferred from these Studies Regarding their Protective Mode of Action?

### A Higher Expression of cDC1 Gene Signatures in Tumors Correlates With a Better Clinical Outcome

#### State-of-the-Art in Assessing cDC1 Infiltration From Whole Tumor Tissue Gene Expression Profiles

Several public datasets are available with gene expression profiles of whole tumor tissue from large cohorts of patients with well documented clinical characteristics. Increasing numbers of teams are querying this gold mine to test whether higher expression in tumors of gene signatures specific for various cell types or biological pathways are associated with a better or worse clinical outcome. Such analyses could allow high throughput testing of the possible relationship between overall survival and tumor infiltration by specific cell types in a given activation state. Such analyses would then allow focusing further studies on the most promising observations, to test whether they are confirmed by using immunohistofluorescence or flow cytometry to directly measure the frequency of specific combinations of immune cell types and activation states in the tumors. However, there is currently no consensus on which gene signatures are the most specific and robust for each immune cell type of interest. In particular, until very recently, to assess the prognostic value of DC infiltration into the tumors, the gene signatures used were those from *in vitro* derived MoDCs. The extent of DC infiltration into tumors as computationally inferred in these studies had no significant prognostic value for overall patient survival, or was even associated to an increased hazard risk (177–181). However, based on the known major differences between MoDCs and cDCs (14–16) and on the beneficial role of mouse cDC1 in antitumor immunity, further studies were needed to assess whether higher infiltration of human tumor by other DC types, in particular cDC1, could be associated with a better clinical outcome.

**Figure 4:**
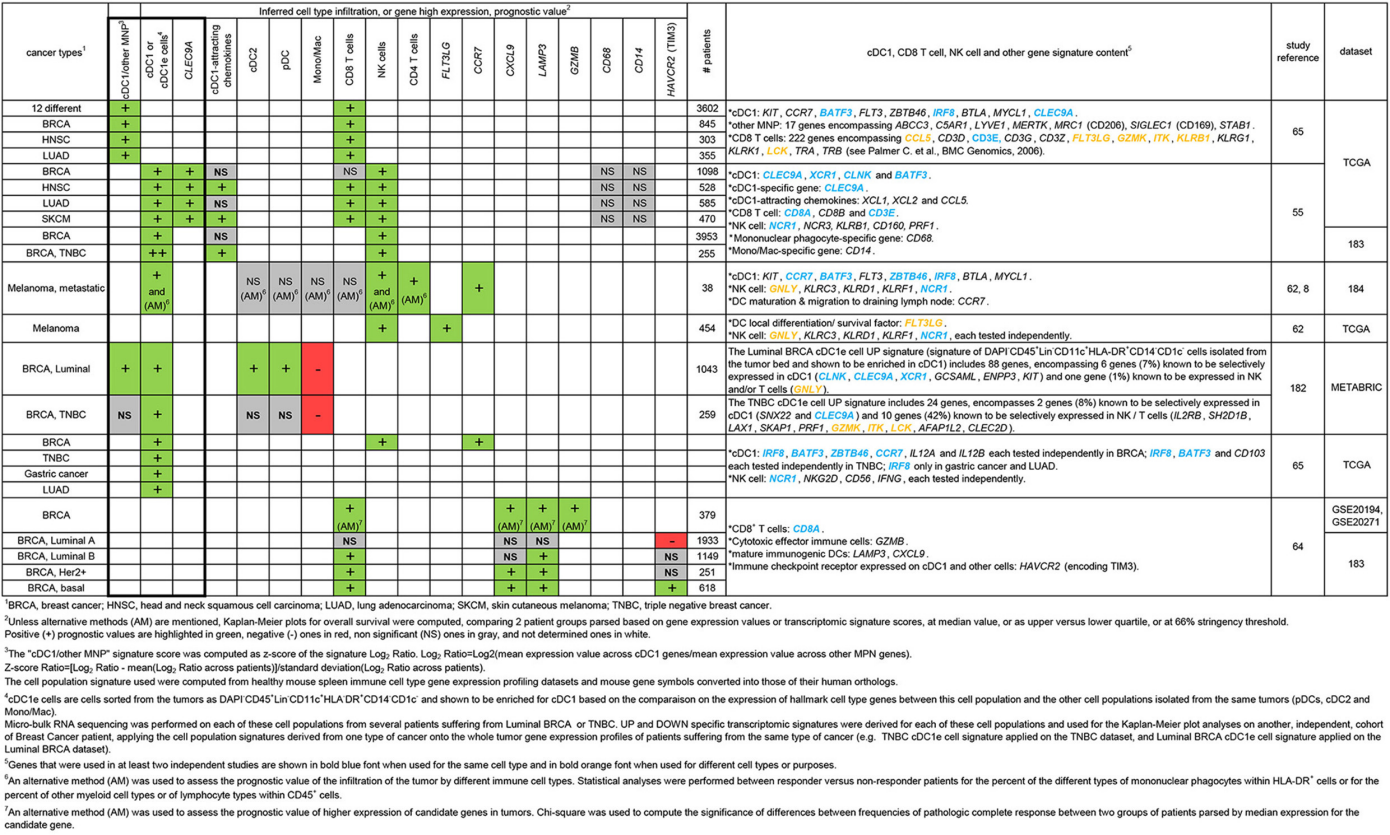
Synthesis of various studies aiming at evaluating the prognosis value of the tumor infiltration by different immune cell types based on the analysis of the whole tumor gene expression profiles.

In the last four years, from the few studies performed to address this issue, a consensus has been emerging that higher expression of cDC1 transcriptomic fingerprints in various tumors correlates with a better clinical outcome (Figure 4, green cells, in the bold rectangle).

In the case for breast cancer (BRCA), a good prognosis of a higher tumor infiltration by cDC1 has been documented independently by 4 studies (55, 65, 66, 182), altogether interrogating three patient cohorts [TCGA, METABRIC, and the meta-cohort generated by Györffy et al. (183)]. A higher expression of the cDC1 transcriptomic signature in tumor was at least as powerful a predictor of prolonged patient survival to cancer as that of the CTL signature (55, 182). Transcriptomic fingerprints or genes associated to certain other immune cell types including cDC2, pDCs or monocytes/macrophages did not have a positive prognostic value (Figure 4, gray or red cells). This supports the hypothesis of a specific protective role of high infiltration of breast tumors by cDC1, rather than the alternate hypothesis that differential levels of cDC1 gene expression in tumors reflect differences in their overall leukocyte infiltration and lead recapitulates the known different clinical outcome of “Hot” or “Warm” vs. “Cold” or “Icy” tumors. However, more studies are warranted to address this issue. For triple negative breast cancer (TNBC), the positive prognostic value of higher cDC1 infiltration in tumors was even better than for all types of BRCA or for luminal BRCA. This was observed in three studies, encompassing altogether the analyses of two patient cohorts (55, 65, 66).

Similar analyses were performed for other types of cancer. For head and neck squamous cell carcinoma (HNSC) and lung adenocarcinoma (LUAD), a higher expression of the cDC1 transcriptomic signature in tumor was also associated to a better clinical outcome by at least two independent studies (55, 62, 80). This was also the case for skin cutaneous melanoma (SKCM) (Figure 4) (47, 72), on two distinct patient cohorts, TCGA and the cohort described by Boguvonic et al. (184). In addition, for metastatic melanoma, the specific positive prognostic value of high cDC1 infiltration in the tumor bed was confirmed by flow cytometry analyses, whereas no significant prognostic value was observed for many other cell types including cDC2, pDCs, Mono/Mac and most surprisingly CTLs (62) (Figure 4). Finally, for LUAD, single cell RNA sequencing and paired CyTOF analyses of tumors and their neighboring normal lung tissue showed that cDC1 were significantly reduced in tumors, contrasting to increased numbers of macrophages in an immunosuppressive state and of cDC2/MoDCs (185). This study further supports the previously proposed hypothesis that the balance between cDC1 vs. cells of the monocyte/macrophage/neutrophil lineages in the tumor leukocyte infiltrate strongly determines the degree of local immunosuppression (65, 93).

#### Limitations, Controversies, or Unknowns

All of the above studies suggest that, in a variety of human cancers, intra-tumoral cDC1 abundance correlates with a better clinical outcome. However, further studies are required to confirm these results, to extend these types of analyses to other types of cancer, and to deepen our understanding of the underlying mechanisms.

A first issue that clearly stands out in Figure 4 is the lack of a consensus definition of the transcriptomic fingerprints used for each immune cell type across studies, not only for cDC1 but even for CTLs or NK cells. Indeed, there is relatively little overlap between the gene signatures used for the same cell types across studies (blue names in Figure 4). Several genes used in some of the cDC1 transcriptomic fingerprints are known to have a promiscuous expression across many cell types (55). *CCR7* and *ITGAE* (CD103) are expressed on all mature DC types and on T cell subsets. *BATF3* and *ZBTB46* are shared with cDC2, and *FLT3* with cDC2, pDCs and hematopoietic progenitors. *IRF8* is highly expressed in pDCs and certain types of monocytes or macrophages. *THBD* (CD141/BDCA3) can be expressed on cDC2, pDCs, MoDCs and non-immune cell types. One study undertook the “tour de force” of profiling by microbulk RNAseq all the distinct mononuclear phagocyte types that they could identify in, and isolate from, BRCA or TNBC, in order to generate the cell-type specific transcriptomic signatures the most relevant to the cancer types studied (182). However, in this study, the TNBC gene signature of the cell population enriched in cDC1 (cDC1e) (182) encompasses only 8% of genes known to be selectively expressed in cDC1 but 42% of genes known to be expressed in NK cells. This raises the question of the interpretation of the positive prognostic value of that signature. It might not only reflect the infiltration of cDC1 but also that of NK cells, in consistency with other analyses in an independent study (55). Indeed, depending on individual samples, the cDC1e population encompassed 50–95% of other cells than cDC1. Using CD16 and CD56 for excluding NK cells from cDC1e cells might have been insufficient, since the strongly activated human NK cells expressing the highest levels of *XCL1* and *XCL2* express neither of these cell surface markers (55). More generally, it is likely that the much higher infiltration of TNBC by lymphocytes, as compared to luminal BRCA, led to major differences in the cell types other than cDC1 that were included in the cDC1e cell population between these two types of cancers. This could confound interpretation of the results of the enrichment analysis of these signatures.

There is a need to define better transcriptomic signatures for human immune cell types, allowing to more rigorously computationally deconvolute the extent of their infiltration in tumors and its eventual correlation with the clinical outcome. One strategy to achieve this aim is to select genes which show high selective expression in the targeted immune cell type across tissues and activation conditions, as well as between human and mouse (14, 45, 153). An alternative strategy could be to perform single cell RNA sequencing from tumor samples, in order to define the transcriptomic signatures specific to various combinations of relevant immune cell types and activation states in the most unbiased way. This strategy would also alleviate the potential confounding effect of cross-contamination between populations as can occur with microbulk gene expression profiling studies (11, 182). Moreover, it will generate transcriptomic signatures specific to the combination of the cell types and of the cancer studied. Indeed, it has been reported that using gene signatures derived from another tissue does not always work adequately to computationally deconvolute the immune cell type composition of tumors, due to differential imprinting of cells in distinct local microenvironments (181, 182).

A second issue is the necessity to include signatures of various types of immune cells, to ensure that the better prognostic value observed in the patients whose tumors harbor higher levels of the genes specific to the candidate immune cell type is not merely a reflect of a higher overall leukocyte infiltration. Indeed, the goal is not just to compare globally “Hot” or “Warm” vs. “Cold” tumors. Rather, it is to pinpoint which immune cell types specifically promote tumor control, or on the contrary contribute to local immunosuppression, in order to identify how to best manipulate the tumor infiltrate, for the benefits of cancer patients, through combined immunotherapies. Thus, the cell types considered to be functionally and/or developmentally the most closely related to the candidate one should be included, for example cDC2 or MoDCs for cDC1, NK cells and γδ T cells for αβ T cells. In addition, one should also include cell types expected to have no, or opposite, impact on tumor growth, for example neutrophils, macrophages and regulatory T cells which are considered as promoting immunosuppression.

A third issue is to deepen our understanding of when, where and how cDC1 promote tumor control. In several studies, the inferred higher cDC1 infiltration in tumors was correlated with higher inferred infiltrations of CTLs or NK cells, and with higher expression of *FLT3L, XCL1, XCL2, CCL4, CCL5, LAMP3, CCR7, CXCL9, CXCL10*, and *CXCL11* (Figure 4) (55, 57, 62, 64, 80, 93). These observations need further independent confirmation through the analysis of other cohorts of patients, and by using complementary methodologies including immunohistochemistry, CyTOF or single cell RNA sequencing to measure the correlation between cDC1 infiltration into the tumors and the status of antitumor NK and CTL responses. In any case, these studies support our proposed model of a critical positive cross talk between cDC1, cytotoxic lymphocytes and CD4^+^ T cells for promoting effective antitumor immunity (Figure 2). Finally, it would be of utmost interest to extend to cohorts of patients benefiting from various types of immunotherapies these analyses aiming at deconvoluting the gene expression profiles of whole tumor tissue into immune cell type composition. This should help determining whether the clinical response can be predicted from cDC1 infiltration in the lesions, and to adapt the treatments accordingly for example by combining to ICB the use of drugs promoting cDC1 recruitment and activation into the tumors of patients when this process is defective (Figure 3).

### Efficacy of Immunotherapeutic Protocols That May Preferentially Target/Harness Human cDC1

A few clinical trials have already been conducted using treatment protocols that have been proposed to preferentially target/harness human cDC1 (Table 6) (186, 187, 190). They gave encouraging results, which further supports the rationale of specifically targeting human cDC1 for the design of novel combined immunotherapies against cancer (189).

**Table 6:** Completed clinical trials targeting cDC1.

**Study start**	**Brief title**	**Condition**	**NCT identifier**	**Intervention**	**Phase**	**Results**	**References**
2006	Peritumoral injection of CpG B with or without GM-CSF for treating patients with stage II Melanoma	Stage II melanoma, planned to undergo sentinel lymph node procedure	Not applicable	Preoperative local injection of either: •GM-CSF + CpG B •CpG B •saline around primary tumor excision site	II	Combined CpG/GM-CSF administration selectively increased cDC1 frequencies and cross-presenting capacity in SLN. cDC1 matured locally upon instruction by GM-CSF and pDCs type I IFN. CpG induced Th1 skewing and increased NK cell and antitumor CTL frequencies in SLN. Higher IL-10 production and Treg activity in SLN. Decreased metastasis in SLN from patients who received CpG.	(186, 187)
2009	A study of CDX-1401 (DEC205/NY-ESO-1) in patients with malignancies known to express NY-ESO-1	Advanced malignancies refractory to available therapies	NCT00948961	CDX-1401 + Resiquimod ± Poly(IC:LC)	I/II	Induction of humoral and cellular immunity to NY-ESO-1. Disease stabilization in 13 of 45 patients. Tumor regression in 2 patients. Objective tumor regression in 6 of 8 patients who received ICB after CDX-1401.	(185)
2014	CDX-1401 (DEC205/NY-ESO-1) and Poly(IC:LC) vaccine therapy with or without CDX-301 in treating patients with stage IIB–IV melanoma	Resected melanoma	NCT02129075	CDX-1401 + Poly(IC:LC) ± rhuFLT3-L (CDX-301) pre-treatment	II	Higher tumor-specific immune responses observed in subjects who received FLT3-L	(188, 189)

*SLN, Sentinel lymph nodes*.

## What Tools are Available to Study and Manipulate Human cDC1 for the Benefits of Cancer Patients?

Building on the conservation of cDC1 molecular makeup and functions between mouse and human, similar tools have been generated in both species to specifically target these cells for immunotherapy against cancer. This should accelerate translation from mouse preclinical studies to human clinical trials. Hence, most of the tools and approaches that have been detailed in the section on mouse experimental models (Figure 3 and Tables 3, 4) could be implemented in humans, as briefly summarized below.

### Generation and Study of Novel *in vitro* Models of Human DC Types

To overcome the roadblock of the rarity of human cDC1 and of their fragility upon *ex vivo* isolation, we and others recently developed optimized *in vitro* culture systems to generate high numbers of cDC1, cDC2 and pDCs from CD34^+^ hematopoietic progenitors (15, 147, 148). These novel *in vitro* models will allow rigorous comparison of the functions of the different human DC types, dissection of their molecular regulation, and better understanding of their cross talk. Further adaptations of these protocols are warranted to derive *in vitro* autologous cDC1 from the circulating CD34^+^ cells of patients, load them with Ag and mature them, under conditions compatible for clinical use in vaccination or immunotherapy. It should be noted that encouraging results have been obtained with clinical trials of autologous ACT of *ex vivo* loaded and matured pDCs and cDC2 in melanoma patients, which seem superior to MoDCs to prime or boost endogenous CTL responses against the tumor. This emphasizes that, as in mice, cDC1 are not the only DC type that could be successfully harnessed for combined immunotherapy in cancer patients (6, 7, 191, 192).

### Means to Specifically Deliver Ag and Maturation Signals to Human cDC1

Considering their conserved specific expression pattern on mouse and human cDC1, and the very encouraging results obtained in mouse preclinical models, the CLEC9A and XCR1 receptors are the best candidates for Ag, or Ag+adjuvant cargo, delivery to human cDC1, using recombinant ligands (193, 194) or monoclonal antibodies. A combination of TLR3- and TLR8-specific agonists is desirable to promote an immunogenic maturation associated with the production of both IL-12 and IFN-β/λ (Table 6) (195). Targeting delivery of IFN-I activity to cDC1 is another very promising adjuvant based on the proof-of-principle published in mice (Tables 3, 5). Additional means could be envisioned to favor the cross-talk between cDC1 and NK or NK T cells (196), e.g., use of NK cell immune checkpoint blockers (101–105) or targeted delivery to cDC1 of activating antigenic ligands for NK T cells (120).

### Means to Promote cDC1 Differentiation, Survival and Local Recruitment in the Tumor Bed

Systemic injection of FLT3-L could promote cDC1 differentiation and survival (79). Local delivery of XCL1 could promote their recruitment in the tumor bed. In patients responding to checkpoint blockade inhibitors, these functions might be achieved upon local NK and CTL activation for FLT3-L, XCL1, and CCL4/5 production (55).

### Blockade of cDC1 Checkpoint Inhibitors

A systematic analysis of immune factor checkpoint expression on human DC types is ongoing in order to investigate which ones could be reasonable candidates as components of combined immunotherapies targeting both CTLs and DCs (197).

## Concluding Remarks

Lately, cDC1 have been in the spotlight of many studies investigating in mice the immune mechanisms driving tumor rejection, spontaneously or upon immunotherapy. All these studies converge toward a hub role of cDC1 in providing the initial priming, or in sustaining the activation, of antitumor T and NK cell responses. These advancements in our understanding of the role of cDC1 in antitumor immunity have been made possible by the recent blossoming of genetic tools allowing cDC1 manipulation. However, so far, most conclusions have been drawn from results obtained under experimental conditions that were not solely targeting cDC1, whether it was the use of genetically engineered mouse models or of mAb directing against cell surface markers. In fact, to be protective against immunosuppressive tumors such as those treated in the clinic, the immune response is necessarily complex and multi-parametric. More and more observations pinpoint that, in addition to cDC1, other DCs, type 1 CD4^+^ T cells, and sometimes neutrophils are also central in promoting protective antitumor immunity, whereas Treg or type 17 CD4^+^ T cells, monocytes and macrophages may rather play immunosuppressive roles. Further studies using models allowing conditional depletion of cDC1 will be critical in rigorously investigating whether cDC1 functions are instrumental at the time when immunotherapies are delivered. These studies will definitely settle the currently prevailing hypothesis that cDC1 functions, when specifically boosted, could provide great support to boost patient responses to currently used anticancer immunotherapies.

In human tumors, enrichment of genetic signatures described as cDC1-specific is associated with a good prognosis and a better clinical outcome in a several cancers, including luminal and TN breast cancer. These correlative analyses should be extended to additional types of cancer and to different patient treatment regimen. It is possible that the extent of cDC1 infiltration in the tumor fluctuates over time following the development or the suppression of an efficient antitumor immune response, as observed in mice during immunotherapy (86), and that cDC1 infiltration may not be protective against all types of cancer. Still, the perspective of exploiting cDC1 to improve current immunotherapies is extremely encouraging, and completion of cDC1-targeting vaccine clinical trials in human will surely help in gaining insight into their importance in cancer.

## Author Contributions

All authors wrote the manuscript, contributed to manuscript revision, read and approved the submitted version.

### Conflict of Interest Statement

The authors declare that the research was conducted in the absence of any commercial or financial relationships that could be construed as a potential conflict of interest.

## References

[1] Quezada SA Peggs KS Simpson TR Allison JP. Shifting the equilibrium in cancer immunoediting: from tumor tolerance to eradication. Immunol Rev. (2011) 241:104–18. 10.1111/j.1600-065X.2011.01007.x 21488893 PMC3727276

[2] Schreiber RD Old LJ Smyth MJ. Cancer immunoediting: integrating immunity's roles in cancer suppression and promotion. Science (2011) 331:1565–70. 10.1126/science.1203486 21436444

[3] Zitvogel L Ma Y Raoult D Kroemer G Gajewski TF. The microbiome in cancer immunotherapy: Diagnostic tools and therapeutic strategies. Science (2018) 359:1366–70. 10.1126/science.aar6918 29567708

[4] Spranger S Gajewski T. Rational combinations of immunotherapeutics that target discrete pathways. J Immunother Cancer (2013) 1:16. 10.1186/2051-1426-1-16 24829752 PMC4019905

[5] Tacken PJ de Vries IJM Torensma R Figdor CG. Dendritic-cell immunotherapy: from *ex vivo* loading to *in vivo* targeting. Nat Rev Immunol. (2007) 7:790–802. 10.1038/nri2173 17853902

[6] Wimmers F Schreibelt G Sköld AE Figdor CG De Vries IJM. Paradigm shift in dendritic cell-based immunotherapy: from *in vitro* generated monocyte-derived DCs to naturally circulating DC subsets. Front Immunol. (2014) 5:165. 10.3389/fimmu.2014.00165 24782868 PMC3990057

[7] Bakdash G Buschow SI Gorris MAJ Halilovic A Hato SV Sköld AE. Expansion of a BDCA1+CD14+ myeloid cell population in melanoma patients may attenuate the efficacy of dendritic cell vaccines. Cancer Res. (2016) 76:4332–46. 10.1158/0008-5472.CAN-15-1695 27325645

[8] Garg AD Coulie PG Van den Eynde BJ Agostinis P. Integrating next-generation dendritic cell vaccines into the current cancer immunotherapy landscape. Trends Immunol. (2017) 38:577–93. 10.1016/j.it.2017.05.006 28610825

[9] Guilliams M Henri S Tamoutounour S Ardouin L Schwartz-Cornil I Dalod M. From skin dendritic cells to a simplified classification of human and mouse dendritic cell subsets. Eur J Immunol. (2010) 40:2089–94. 10.1002/eji.201040498 20853491

[10] Guilliams M Ginhoux F Jakubzick C Naik SH Onai N Schraml BU. Dendritic cells, monocytes and macrophages: a unified nomenclature based on ontogeny. Nat Rev Immunol. (2014) 14:571–8. 10.1038/nri3712 25033907 PMC4638219

[11] Vu Manh T-P Bertho N Hosmalin A Schwartz-Cornil I Dalod M Investigating evolutionary conservation of dendritic cell subset identity and functions. Front Immunol. (2015) 6:260 10.3389/fimmu.2015.00260 26082777 PMC4451681

[12] Schlitzer A Sivakamasundari V Chen J Sumatoh HRB Schreuder J Lum J. Identification of cDC1- and cDC2-committed DC progenitors reveals early lineage priming at the common DC progenitor stage in the bone marrow. Nat Immunol. (2015) 16:718–28. 10.1038/ni.3200 26054720

[13] See P Dutertre C-A Chen J Günther P McGovern N Irac SE. Mapping the human DC lineage through the integration of high-dimensional techniques. Science (2017) 356:eaag3009. 10.1126/science.aag3009 28473638 PMC7611082

[14] Robbins SH Walzer T Dembélé D Thibault C Defays A Bessou G. Novel insights into the relationships between dendritic cell subsets in human and mouse revealed by genome-wide expression profiling. Genome Biol. (2008) 9:R17. 10.1186/gb-2008-9-1-r17 18218067 PMC2395256

[15] Balan S Ollion V Colletti N Chelbi R Montanana-Sanchis F Liu H. Human XCR1+ dendritic cells derived *in vitro* from CD34+ progenitors closely resemble blood dendritic cells, including their adjuvant responsiveness, contrary to monocyte-derived dendritic cells. J Immunol. (2014) 193:1622–35. 10.4049/jimmunol.1401243 25009205 PMC4120898

[16] Alcántara-Hernández M Leylek R Wagar LE Engleman EG Keler T Marinkovich MP. High-Dimensional phenotypic mapping of human dendritic cells reveals interindividual variation and tissue specialization. Immunity (2017) 47:1037–50.e6. 10.1016/j.immuni.2017.11.001 29221729 PMC5738280

[17] Fries A Dalod M Dendritic Cells in Viral Infection in Encyclopedia of Immunobiology, ed Ratcliffe M. J. H. (Oxford: Academic Press), 207–221.

[18] van Blijswijk J Schraml BU Reis e Sousa C Advantages and limitations of mouse models to deplete dendritic cells. Eur J Immunol. (2013) 43:22–6. 10.1002/eji.201243022 23322690

[19] Durai V Murphy KM. Functions of murine dendritic cells. Immunity (2016) 45:719–36. 10.1016/j.immuni.2016.10.010 27760337 PMC5145312

[20] Jung S Unutmaz D Wong P Sano G-I De los Santos K Sparwasser T. *In vivo* depletion of CD11c+ dendritic cells abrogates priming of CD8^+^ T cells by exogenous cell-associated antigens. Immunity (2002) 17:211–20. 10.1016/S1074-7613(02)00365-5 12196292 PMC3689299

[21] Sapoznikov A Fischer JAA Zaft T Krauthgamer R Dzionek A Jung S Organ-dependent *in vivo* priming of naive CD4+, but not CD8^+^, T cells by plasmacytoid dendritic cells. J Exp Med. (2007) 204:1923–33. 10.1084/jem.20062373 17646404 PMC2118686

[22] Probst HC Tschannen K Odermatt B Schwendener R Zinkernagel RM Van Den Broek M. Histological analysis of CD11c-DTR/GFP mice after *in vivo* depletion of dendritic cells. Clin Exp Immunol. (2005) 141:398–404. 10.1111/j.1365-2249.2005.02868.x 16045728 PMC1809468

[23] van Rijt LS Jung S Kleinjan A Vos N Willart M Duez C *In vivo* depletion of lung CD11c+ dendritic cells during allergen challenge abrogates the characteristic features of asthma. J Exp Med. (2005) 201:981–91. 10.1084/jem.20042311 15781587 PMC2213109

[24] Vallon-Eberhard A Landsman L Yogev N Verrier B Jung S. Transepithelial pathogen uptake into the small intestinal lamina propria. J Immunol. (2006) 176:2465–9. 10.4049/jimmunol.176.4.2465 16456006

[25] Huleatt JW Lefrançois L. Antigen-driven induction of CD11c on intestinal intraepithelial lymphocytes and CD8^+^ T cells *in vivo*. J Immunol. (1995) 154:5684–93. 7751620

[26] Laouar Y Sutterwala FS Gorelik L Flavell RA. Transforming growth factor-beta controls T helper type 1 cell development through regulation of natural killer cell interferon-gamma. Nat Immunol. (2005) 6:600–7. 10.1038/ni1197 15852008

[27] Hebel K Griewank K Inamine A Chang H-D Müller-Hilke B Fillatreau S Manz RA Radbruch A Jung S. Plasma cell differentiation in T-independent type 2 immune responses is independent of CD11c(high) dendritic cells. Eur J Immunol. (2006) 36:2912–9. 10.1002/eji.200636356 17051619

[28] Hochweller K Striegler J Hämmerling GJ Garbi N. A novel CD11c.DTR transgenic mouse for depletion of dendritic cells reveals their requirement for homeostatic proliferation of natural killer cells. Eur J Immunol. (2008) 38:2776–83. 10.1002/eji.200838659 18825750

[29] Fukaya T Murakami R Takagi H Sato K Sato Y Otsuka H. Conditional ablation of CD205+ conventional dendritic cells impacts the regulation of T-cell immunity and homeostasis *in vivo*. Proc Natl Acad Sci USA. (2012) 109:11288–93. 10.1073/pnas.1202208109 22736794 PMC3396526

[30] Laoui D Keirsse J Morias Y Van Overmeire E Geeraerts X Elkrim Y. The tumour microenvironment harbours ontogenically distinct dendritic cell populations with opposing effects on tumour immunity. Nat Commun. (2016) 7:13720. 10.1038/ncomms13720 28008905 PMC5196231

[31] Fukaya T Takagi H Uto T Arimura K Sato K. Analysis of DC Functions Using CD205-DTR Knock-In Mice. Methods Mol Biol. (2016) 1423:291–308. 10.1007/978-1-4939-3606-9_21 27142025

[32] Satpathy AT Kc W Albring JC Edelson BT Kretzer NM Bhattacharya D. Zbtb46 expression distinguishes classical dendritic cells and their committed progenitors from other immune lineages. J Exp Med. (2012) 209:1135–52. 10.1084/jem.20120030 22615127 PMC3371733

[33] Meredith MM Liu K Darrasse-Jeze G Kamphorst AO Schreiber HA Guermonprez P. Expression of the zinc finger transcription factor zDC (Zbtb46, Btbd4) defines the classical dendritic cell lineage. J Exp Med. (2012) 209:1153–65. 10.1084/jem.20112675 22615130 PMC3371731

[34] Loschko J Rieke GJ Schreiber HA Meredith MM Yao K-H Guermonprez P. Inducible targeting of cDCs and their subsets *in vivo*. J Immunol Methods (2016) 434:32–8. 10.1016/j.jim.2016.04.004 27073171 PMC4902770

[35] Hildner K Edelson BT Purtha WE Diamond M Matsushita H Kohyama M. Batf3 deficiency reveals a critical role for CD8alpha+ dendritic cells in cytotoxic T cell immunity. Science (2008) 322:1097–100. 10.1126/science.1164206 19008445 PMC2756611

[36] Lee W Kim HS Hwang SS Lee GR. The transcription factor Batf3 inhibits the differentiation of regulatory T cells in the periphery. Exp Mol Med. (2017) 49:e393. 10.1038/emm.2017.157 29147008 PMC5704186

[37] Edelson BT Bradstreet TR Kc W Hildner K Herzog JW Sim J. Batf3-dependent CD11b(low/-) peripheral dendritic cells are GM-CSF-independent and are not required for Th cell priming after subcutaneous immunization. PLoS ONE (2011) 6:e25660. 10.1371/journal.pone.0025660 22065991 PMC3196467

[38] Tussiwand R Lee W-L Murphy TL Mashayekhi M Kc W Albring JC. Compensatory dendritic cell development mediated by BATF-IRF interactions. Nature (2012) 490:502–7. 10.1038/nature11531 22992524 PMC3482832

[39] Grajales-Reyes GE Iwata A Albring J Wu X Tussiwand R Kc W Kretzer NM. Batf3 maintains autoactivation of Irf8 for commitment of a CD8α(+) conventional DC clonogenic progenitor. Nat Immunol. (2015) 16:708–17. 10.1038/ni.3197 26054719 PMC4507574

[40] Iacobelli M Wachsman W McGuire KL. Repression of IL-2 promoter activity by the novel basic leucine zipper p21SNFT protein. J Immunol. (2000) 165:860–8. 10.4049/jimmunol.165.2.860 10878360

[41] Piva L Tetlak P Claser C Karjalainen K Renia L Ruedl C. Cutting edge: Clec9A+ dendritic cells mediate the development of experimental cerebral malaria. J Immunol. (2012) 189:1128–32. 10.4049/jimmunol.1201171 22732587

[42] Mattiuz R Wohn C Ghilas S Ambrosini M Alexandre YO Sanchez C. Novel Cre-expressing mouse strains permitting to selectively track and edit type 1 conventional dendritic cells facilitate disentangling their complexity *in vivo*. Front Immunol. (2018) 9:2805 10.3389/fimmu.2018.02805 30564233 PMC6288293

[43] Alexandre YO Ghilas S Sanchez C Le Bon A Crozat K Dalod M XCR1^+^ dendritic cells promote memory CD8^+^ T cell recall upon secondary infections with *Listeria monocytogenes* or certain viruses. J Exper Med. (2016) 213:75–92. 10.1084/jem.20142350 26694969 PMC4710197

[44] Yamazaki C Sugiyama M Ohta T Hemmi H Hamada E Sasaki I. Critical roles of a dendritic cell subset expressing a chemokine receptor, XCR1. J Immunol. (2013) 190:6071–82. 10.4049/jimmunol.1202798 23670193

[45] Crozat K Guiton R Contreras V Feuillet V Dutertre C-A Ventre E. The XC chemokine receptor 1 is a conserved selective marker of mammalian cells homologous to mouse CD8alpha+ dendritic cells. J Exp Med. (2010) 207:1283–92. 10.1084/jem.20100223 20479118 PMC2882835

[46] Ohta T Sugiyama M Hemmi H Yamazaki C Okura S Sasaki I. Crucial roles of XCR1-expressing dendritic cells and the XCR1-XCL1 chemokine axis in intestinal immune homeostasis. Sci Reports (2016) 6:23505. 10.1038/srep23505 27005831 PMC4804307

[47] Schraml BU van Blijswijk J Zelenay S Whitney PG Filby A Acton SE. Genetic tracing via DNGR-1 expression history defines dendritic cells as a hematopoietic lineage. Cell (2013) 154:843–58. 10.1016/j.cell.2013.07.014 23953115

[48] Hanahan D Weinberg RA. Hallmarks of cancer: the next generation. Cell (2011) 144:646–74. 10.1016/j.cell.2011.02.013 21376230

[49] Dunn GP Old LJ Schreiber RD. The three Es of cancer immunoediting. Annu Rev Immunol. (2004) 22:329–60. 10.1146/annurev.immunol.22.012703.104803 15032581

[50] Vesely MD Kershaw MH Schreiber RD Smyth MJ. Natural innate and adaptive immunity to cancer. Annu Rev Immunol. (2011) 29:235–71. 10.1146/annurev-immunol-031210-101324 21219185

[51] Diamond MS Kinder M Matsushita H Mashayekhi M Dunn GP Archambault JM. Type I interferon is selectively required by dendritic cells for immune rejection of tumors. J Exp Med. (2011) 208:1989–2003. 10.1084/jem.20101158 21930769 PMC3182061

[52] Theisen DJ Davidson JT Briseño CG Gargaro M Lauron EJ Wang Q. WDFY4 is required for cross-presentation in response to viral and tumor antigens. Science (2018) 362:694–9. 10.1126/science.aat5030 30409884 PMC6655551

[53] Theisen DJ Ferris ST Briseño CG Kretzer N Iwata A Murphy KM. BATF3-dependent genes control tumor rejection induced by dendritic cells independently of cross-presentation. Cancer Immunol Res. (2018). 7:29–39. 10.1158/2326-6066.CIR-18-0138 30482745 PMC12093468

[54] Fuertes MB Kacha AK Kline J Woo SR Kranz DM Murphy KM. Host type I IFN signals are required for antitumor CD8^+^ T cell responses through CD8{alpha}+ dendritic cells. J Exper Med. (2011) 208:2005–16. 10.1084/jem.20101159 21930765 PMC3182064

[55] Bottcher JP Bonavita E Chakravarty P Blees H Cabeza-Cabrerizo M Sammicheli S Rogers NC Sahai E Zelenay S Reis ESC. NK Cells Stimulate Recruitment of cDC1 into the Tumor Microenvironment Promoting Cancer Immune Control. Cell (2018) 172:1022–e14. 10.1016/j.cell.2018.01.004 29429633 PMC5847168

[56] Zelenay S van der Veen AG Bottcher JP Snelgrove KJ Rogers N Acton SE Chakravarty P Girotti MR Marais R Quezada SA. Cyclooxygenase-dependent tumor growth through evasion of immunity. Cell (2015) 162:1257–70. 10.1016/j.cell.2015.08.015 26343581 PMC4597191

[57] Spranger S Dai D Horton B Gajewski TF. Tumor-Residing Batf3 Dendritic Cells Are Required for Effector T Cell Trafficking and Adoptive T Cell Therapy. Cancer cell (2017) 31:711–e4. 10.1016/j.ccell.2017.04.003 28486109 PMC5650691

[58] Iraolagoitia XLR Spallanzani RG Torres NI Araya RE Ziblat A Domaica CI. NK cells restrain spontaneous antitumor CD8^+^ T cell priming through PD-1/PD-L1 interactions with dendritic cells. J Immunol. (2016) 197:953–961. 10.4049/jimmunol.1502291 27342842

[59] Steinman RM Idoyaga J. Features of the dendritic cell lineage. Immunol Rev. (2010) 234:5–17. 10.1111/j.0105-2896.2009.00888.x 20193008

[60] Sanchez-Paulete AR Teijeira A Cueto FJ Garasa S Perez-Gracia JL Sanchez-Arraez A Antigen cross-presentation and T-cell cross-priming in cancer immunology and immunotherapy. Ann Oncol. (2017) 28:xii44–55. 10.1093/annonc/mdx237 28945841

[61] Dorner BG Dorner MB Zhou X Opitz C Mora A Guttler S Hutloff A. Selective expression of the chemokine receptor XCR1 on cross-presenting dendritic cells determines cooperation with CD8^+^ T cells. Immunity (2009) 31:823–33. 10.1016/j.immuni.2009.08.027 19913446

[62] Barry KC Hsu J Broz ML Cueto FJ Binnewies M Combes AJ Nelson AE. A natural killer–dendritic cell axis defines checkpoint therapy–responsive tumor microenvironments. Nat Med. (2018) 24:1178–91. 10.1038/s41591-018-0085-8 29942093 PMC6475503

[63] Bachem A Güttler S Hartung E Ebstein F Schaefer M Tannert A. Superior antigen cross-presentation and XCR1 expression define human CD11c+CD141+ cells as homologues of mouse CD8^+^ dendritic cells. J Exp Med. (2010) 207:1273–81. 10.1084/jem.20100348 20479115 PMC2882837

[64] de Mingo Pulido A Gardner A Hiebler S Soliman H Rugo HS Krummel MF TIM-3 Regulates CD103(+) Dendritic cell function and response to chemotherapy in breast cancer. Cancer cell (2018) 33:60–74.e6. 10.1016/j.ccell.2017.11.019 29316433 PMC5764109

[65] Broz ML Binnewies M Boldajipour B Nelson AE Pollack JL Erle DJ Dissecting the tumor myeloid compartment reveals rare activating antigen-presenting cells critical for T cell immunity. Cancer Cell (2014) 26:638–52. 10.1016/j.ccell.2014.09.007 25446897 PMC4254577

[66] Mittal D Vijayan D Putz EM Aguilera AR Markey KA Straube J. Interleukin-12 from CD103(+) Batf3-dependent dendritic cells required for NK-Cell suppression of metastasis. Cancer Immunol Res. (2017) 5:1098–108. 10.1158/2326-6066.CIR-17-0341 29070650

[67] Ruffell B Chang-Strachan D Chan V Rosenbusch A Ho CM Pryer N. Macrophage IL-10 blocks CD8^+^ T cell-dependent responses to chemotherapy by suppressing IL-12 expression in intratumoral dendritic cells. Cancer Cell (2014) 26:623–37. 10.1016/j.ccell.2014.09.006 25446896 PMC4254570

[68] Greyer M Whitney PG Stock AT Davey GM Tebartz C Bachem A. T cell help amplifies innate signals in CD8(+) DCs for optimal CD8(+) T cell priming. Cell Rep. (2016) 14:586–97. 10.1016/j.celrep.2015.12.058 26774484

[69] Beavis PA Henderson MA Giuffrida L Davenport AJ Petley EV House IG. Dual PD-1 and CTLA-4 checkpoint blockade promotes antitumor immune responses through CD4^+^ Foxp3^−^ cell–mediated modulation of CD103 ^+^ dendritic cells. Cancer Immunol Res. (2018) 6:1069–81. 10.1158/2326-6066.CIR-18-0291 30018045

[70] Draheim M Wlodarczyk MF Crozat K Saliou J-M Alayi TD Tomavo S. Profiling MHC II immunopeptidome of blood-stage malaria reveals that cDC1 control the functionality of parasite-specific CD4 T cells. EMBO Mol Med. (2017) 9:1605–21. 10.15252/emmm.201708123 28935714 PMC5666312

[71] Igyártó BZ Haley K Ortner D Bobr A Gerami-Nejad M Edelson BT. Skin-resident murine dendritic cell subsets promote distinct and opposing antigen-specific T helper cell responses. Immunity (2011) 35:260–72. 10.1016/j.immuni.2011.06.005 21782478 PMC3163010

[72] Martínez-López M Iborra S Conde-Garrosa R Sancho D. Batf3-dependent CD103+ dendritic cells are major producers of IL-12 that drive local Th1 immunity against Leishmania major infection in mice. Eur J Immunol. (2015) 45:119–29. 10.1002/eji.201444651 25312824 PMC4316187

[73] Eickhoff S Brewitz A Gerner MY Klauschen F Komander K Hemmi H. Robust Anti-viral Immunity Requires Multiple Distinct T Cell-Dendritic Cell Interactions. Cell (2015) 162:1322–37. 10.1016/j.cell.2015.08.004 26296422 PMC4567961

[74] Hor JL Whitney PG Zaid A Brooks AG Heath WR Mueller SN. Spatiotemporally distinct interactions with dendritic cell subsets facilitates CD4+ and CD8^+^ T cell activation to localized viral infection. Immunity (2015) 43:554–65. 10.1016/j.immuni.2015.07.020 26297566

[75] Ardouin L Luche H Chelbi R Carpentier S Shawket A Montanana Sanchis F. Broad and largely concordant molecular changes characterize tolerogenic and immunogenic dendritic cell maturation in thymus and periphery. Immunity (2016) 45:305–18. 10.1016/j.immuni.2016.07.019 27533013

[76] Dalod M Chelbi R Malissen B Lawrence T. Dendritic cell maturation: functional specialization through signaling specificity and transcriptional programming. EMBO J. (2014) 33:1104–116. 10.1002/embj.201488027 24737868 PMC4193918

[77] Brewitz A Eickhoff S Dähling S Quast T Bedoui S Kroczek RA. CD8^+^ T Cells Orchestrate pDC-XCR1+ dendritic cell spatial and functional cooperativity to optimize priming. Immunity (2017) 46:205–19. 10.1016/j.immuni.2017.01.003 28190711 PMC5362251

[78] Enamorado M Iborra S Priego E Cueto FJ Quintana JA Martinez-Cano S. Enhanced anti-tumour immunity requires the interplay between resident and circulating memory CD8(+) T cells. Nat Commun. (2017) 8:16073. 10.1038/ncomms16073 28714465 PMC5520051

[79] Salmon H Idoyaga J Rahman A Leboeuf M Remark R Jordan S. Expansion and activation of CD103(+) dendritic cell progenitors at the tumor site enhances tumor responses to therapeutic PD-L1 and BRAF inhibition. Immunity (2016) 44:924–38. 10.1016/j.immuni.2016.03.012 27096321 PMC4980762

[80] Roberts EW Broz ML Binnewies M Headley MB Nelson AE Wolf DM. Critical role for CD103(+)/CD141(+) dendritic cells bearing CCR7 for tumor antigen trafficking and priming of T cell immunity in melanoma. Cancer Cell (2016) 30:324–36. 10.1016/j.ccell.2016.06.003 27424807 PMC5374862

[81] Matloubian M Lo CG Cinamon G Lesneski MJ Xu Y Brinkmann V. Lymphocyte egress from thymus and peripheral lymphoid organs is dependent on S1P receptor 1. Nature (2004) 427:355–60. 10.1038/nature02284 14737169

[82] Thompson ED Enriquez HL Fu Y-X Engelhard VH. Tumor masses support naive T cell infiltration, activation, and differentiation into effectors. J Exper Med (2010) 207:1791–804. 10.1084/jem.20092454 20660615 PMC2916130

[83] Lan YY De Creus A Colvin BL Abe M Brinkmann V Coates PTH. The sphingosine-1-phosphate receptor agonist FTY720 modulates dendritic cell trafficking in vivo. Am J Transp. (2005) 5:2649–59. 10.1111/j.1600-6143.2005.01085.x 16212624

[84] Morris MA Gibb DR Picard F Brinkmann V Straume M Ley K. Transient T cell accumulation in lymph nodes and sustained lymphopenia in mice treated with FTY720. Eur J Immunol. (2005) 35:3570–80. 10.1002/eji.200526218 16285007

[85] Dieu-Nosjean M-C Giraldo NA Kaplon H Germain C Fridman WH Sautès-Fridman C. Tertiary lymphoid structures, drivers of the anti-tumor responses in human cancers. Immunol Rev. (2016) 271:260–75. 10.1111/imr.12405 27088920

[86] Spitzer MH Carmi Y Reticker-Flynn NE Kwek SS Madhireddy D Martins MM. Systemic immunity is required for effective cancer immunotherapy. Cell (2017) 168:487–502.e15. 10.1016/j.cell.2016.12.022 28111070 PMC5312823

[87] Spranger S Gajewski TF Mechanisms of tumor cell–intrinsic immune evasion. Ann Rev Cancer Biol. (2018) 2:213–28. 10.1146/annurev-cancerbio-030617-050606

[88] Paulson KG Voillet V McAfee MS Hunter DS Wagener FD Perdicchio M. Acquired cancer resistance to combination immunotherapy from transcriptional loss of class I HLA. Nat Commun. (2018) 9:3868. 10.1038/s41467-018-06300-3 30250229 PMC6155241

[89] Pauken KE Wherry EJ. Overcoming T cell exhaustion in infection and cancer. Trends Immunol. (2015) 36:265–76. 10.1016/j.it.2015.02.008 25797516 PMC4393798

[90] Zong J Keskinov AA Shurin GV Shurin MR. Tumor-derived factors modulating dendritic cell function. Cancer Immunol Immunother. (2016) 65:821–33. 10.1007/s00262-016-1820-y 26984847 PMC11028482

[91] Perry JSA Lio C-WJ Kau AL Nutsch K Yang Z Gordon JI. Distinct contributions of Aire and antigen-presenting-cell subsets to the generation of self-tolerance in the thymus. Immunity (2014) 41:414–26. 10.1016/j.immuni.2014.08.007 25220213 PMC4175925

[92] Esterházy D Loschko J London M Jove V Oliveira TY Mucida D. Classical dendritic cells are required for dietary antigen-mediated induction of peripheral T(reg) cells and tolerance. Nat Immunol. (2016) 17:545–55. 10.1038/ni.3408 27019226 PMC4837106

[93] Spranger S Bao R Gajewski TF. Melanoma-intrinsic beta-catenin signalling prevents anti-tumour immunity. Nature (2015) 523:231–5. 10.1038/nature14404 25970248

[94] Cook SJ Lee Q Wong AC Spann BC Vincent JN Wong JJ. Differential chemokine receptor expression and usage by pre-cDC1 and pre-cDC2. Immunol Cell Biol. (2018) 96:1131–9 10.1111/imcb.12186 29920767

[95] Fucikova J Becht E Iribarren K Goc J Remark R Damotte D. Calreticulin expression in human non-small cell lung cancers correlates with increased accumulation of antitumor immune cells and favorable prognosis. Cancer Res. (2016) 76:1746–56. 10.1158/0008-5472.CAN-15-1142 26842877

[96] Garg AD Dudek AM Ferreira GB Verfaillie T Vandenabeele P Krysko DV. ROS-induced autophagy in cancer cells assists in evasion from determinants of immunogenic cell death. Autophagy (2013) 9:1292–307. 10.4161/auto.25399 23800749

[97] Balogh KN Templeton DJ Cross JV. Macrophage Migration Inhibitory Factor protects cancer cells from immunogenic cell death and impairs anti-tumor immune responses. PLoS ONE (2018) 13:e0197702. 10.1371/journal.pone.0197702 29864117 PMC5986154

[98] Meyer MA Baer JM Knolhoff BL Nywening TM Panni RZ Su X. Breast and pancreatic cancer interrupt IRF8-dependent dendritic cell development to overcome immune surveillance. Nature Commun. (2018) 9:1250. 10.1038/s41467-018-03600-6 29593283 PMC5871846

[99] Veglia F Tyurin VA Mohammadyani D Blasi M Duperret EK Donthireddy L. Lipid bodies containing oxidatively truncated lipids block antigen cross-presentation by dendritic cells in cancer. Nat Commun. (2017) 8:2122. 10.1038/s41467-017-02186-9 29242535 PMC5730553

[100] Kline DE MacNabb BW Chen X Chan W-C Fosco D Kline J. CD8α+ dendritic cells dictate leukemia-specific CD8^+^ T cell fates. J Immunol. (2018) 201:3759–69. 10.4049/jimmunol.1801184 30420437 PMC6444187

[101] Hsu J Hodgins JJ Marathe M Nicolai CJ Bourgeois-Daigneault M-C Trevino TN. Contribution of NK cells to immunotherapy mediated by PD-1/PD-L1 blockade. J Clin Invest. (2018) 128:4654–68. 10.1172/JCI99317 30198904 PMC6159991

[102] Zhang Q Bi J Zheng X Chen Y Wang H Wu W. Blockade of the checkpoint receptor TIGIT prevents NK cell exhaustion and elicits potent anti-tumor immunity. Nat Immunol. (2018) 19:723–32. 10.1038/s41590-018-0132-0 29915296

[103] Kohrt HE Thielens A Marabelle A Sagiv-Barfi I Sola C Chanuc F. Anti-KIR antibody enhancement of anti-lymphoma activity of natural killer cells as monotherapy and in combination with anti-CD20 antibodies. Blood (2014) 123:678–86. 10.1182/blood-2013-08-519199 24326534 PMC3907754

[104] Sola C André P Lemmers C Fuseri N Bonnafous C Bléry M. Genetic and antibody-mediated reprogramming of natural killer cell missing-self recognition *in vivo*. Proc Natl Acad Sci USA. (2009) 106:12879–84. 10.1073/pnas.0901653106 19561305 PMC2722344

[105] Chiossone L Dumas P-Y Vienne M Vivier E Natural killer cells and other innate lymphoid cells in cancer. Nat Rev Immunol. (2018) 18:671–88. 10.1038/s41577-018-0061-z 30209347

[106] Pardoll DM. The blockade of immune checkpoints in cancer immunotherapy. Nat Rev Cancer (2012) 12:252–64. 10.1038/nrc3239 22437870 PMC4856023

[107] Farkona S Diamandis EP Blasutig IM. Cancer immunotherapy: the beginning of the end of cancer? BMC Med. (2016) 14:73. 10.1186/s12916-016-0623-5 27151159 PMC4858828

[108] Larkin J Chiarion-Sileni V Gonzalez R Grob JJ Cowey CL Lao CD Combined nivolumab and ipilimumab or monotherapy in untreated melanoma. N Engl J Med. (2015) 373:23–34. 10.1056/NEJMoa1504030 26027431 PMC5698905

[109] Postow MA Chesney J Pavlick AC Robert C Grossmann K McDermott D. Nivolumab and Ipilimumab versus Ipilimumab in Untreated Melanoma. N Engl J Med. (2015) 372:2006–17. 10.1056/NEJMoa1414428 25891304 PMC5744258

[110] Diao J Gu H Tang M Zhao J Cattral MS. Tumor dendritic cells (DCs) derived from precursors of conventional DCs are dispensable for intratumor CTL responses. J Immunol (2018) 201:1306–14. 10.4049/jimmunol.1701514 29997124 PMC6077850

[111] Yu X Guo C Yi H Qian J Fisher PB Subjeck JR Wang XY. A multifunctional chimeric chaperone serves as a novel immune modulator inducing therapeutic antitumor immunity. Cancer Res. (2013) 73:2093–103. 10.1158/0008-5472.can-12-1740 23333935 PMC3618619

[112] Yang X Zhang X Fu ML Weichselbaum RR Gajewski TF Guo Y. Targeting the tumor microenvironment with interferon-beta bridges innate and adaptive immune responses. Cancer Cell (2014) 25:37–48. 10.1016/j.ccr.2013.12.004 24434209 PMC3927846

[113] Zhang Y Chen G Liu Z Tian S Zhang J Carey CD. Genetic vaccines to potentiate the effective CD103+ dendritic cell-mediated cross-priming of antitumor immunity. J Immunol (Baltimore, Md: 1950) (2015) 194:5937–43. 10.4049/jimmunol.1500089 25972487 PMC4458448

[114] Tzeng A Kauke MJ Zhu EF Moynihan KD Opel CF Yang NJ. Temporally programmed CD8alpha(+) DC activation enhances combination cancer immunotherapy. Cell Reports (2016) 17:2503–11. 10.1016/j.celrep.2016.11.020 27926855 PMC5204262

[115] Cauwels A Van Lint S Paul F Garcin G De Koker S Van Parys A. Delivering type I interferon to dendritic cells empowers tumor eradication and immune combination treatments. Cancer Res. (2018) 78:463–74. 10.1158/0008-5472.CAN-17-1980 29187401

[116] Gilfillan CB Kuhn S Baey C Hyde EJ Yang J Ruedl C Clec9A^+^ dendritic cells are not essential for antitumor CD8^+^ T cell responses induced by Poly I:C immunotherapy. J Immunol. (2018) 200:2978–86. 10.4049/jimmunol.1701593 29507107

[117] Gubin MM Zhang X Schuster H Caron E Ward JP Noguchi T. Checkpoint blockade cancer immunotherapy targets tumour-specific mutant antigens. Nature (2014) 515:577–81. 10.1038/nature13988 25428507 PMC4279952

[118] Sanchez-Paulete AR Cueto FJ Martinez-Lopez M Labiano S Morales-Kastresana A Rodriguez-Ruiz ME. Cancer immunotherapy with immunomodulatory Anti-CD137 and Anti-PD-1 monoclonal antibodies requires BATF3-dependent dendritic cells. Cancer Disc. (2016) 6:71–9. 10.1158/2159-8290.cd-15-0510 26493961 PMC5036540

[119] Lehmann C Heger L Heidkamp G Baranska A Lühr J Hoffmann A. Direct delivery of antigens to dendritic cells via antibodies specific for endocytic receptors as a promising strategy for future therapies. Vaccines (2016) 4:8. 10.3390/vaccines4020008 27043640 PMC4931625

[120] Ghinnagow R Meester JD Cruz LJ Aspord C Corgnac S Macho-Fernandez E. Co-delivery of the NKT agonist α-galactosylceramide and tumor antigens to cross-priming dendritic cells breaks tolerance to self-antigens and promotes antitumor responses. OncoImmunology (2017) 6:e1339855. 10.1080/2162402X.2017.1339855 28932640 PMC5599097

[121] Macho-Fernandez E Cruz LJ Ghinnagow R Fontaine J Bialecki E Frisch B. Targeted delivery of α-Galactosylceramide to CD8α+ dendritic cells optimizes type I NKT cell–based antitumor responses. J Immunol. (2014) 193:961–9. 10.4049/jimmunol.1303029 24913977

[122] Filatenkov AA Jacovetty EL Fischer UB Curtsinger JM Mescher MF Ingulli E. CD4 T cell-dependent conditioning of dendritic cells to produce IL-12 results in CD8-mediated graft rejection and avoidance of tolerance. J Immunol. (2005) 174:6909–17. 10.4049/jimmunol.174.11.6909 15905533

[123] Oh S Perera LP Terabe M Ni L Waldmann TA Berzofsky JA. IL-15 as a mediator of CD4+ help for CD8^+^ T cell longevity and avoidance of TRAIL-mediated apoptosis. Proc Natl Acad Sci USA. (2008) 105:5201–6. 10.1073/pnas.0801003105 18362335 PMC2278231

[124] Cao J Jin Y Li W Zhang B He Y Liu H. DNA vaccines targeting the encoded antigens to dendritic cells induce potent antitumor immunity in mice. BMC Immunol. (2013) 14:39. 10.1186/1471-2172-14-39 23941509 PMC3751307

[125] Tenbusch M Nchinda G Storcksdieck genannt Bonsmann M Temchura V Überla K. Targeting the antigen encoded by adenoviral vectors to the DEC205 receptor modulates the cellular and humoral immune response. Int Immunol. (2013) 25:247–58. 10.1093/intimm/dxs112 23184617

[126] Ali OA Verbeke C Johnson C Sands RW Lewin SA White D. Identification of immune factors regulating antitumor immunity using polymeric vaccines with multiple adjuvants. Cancer Res. (2014) 74:1670–81. 10.1158/0008-5472.can-13-0777 24480625 PMC3959905

[127] Zeng B Middelberg AP Gemiarto A MacDonald K Baxter AG Talekar M. Self-adjuvanting nanoemulsion targeting dendritic cell receptor Clec9A enables antigen-specific immunotherapy. J Clin Invest (2018) 128:1971–84. 10.1172/JCI96791 29485973 PMC5919883

[128] Idoyaga J Fiorese C Zbytnuik L Lubkin A Miller J Malissen B. Specialized role of migratory dendritic cells in peripheral tolerance induction. J Clin Investig. (2013) 123:844–54. 10.1172/JCI65260 23298832 PMC3561796

[129] Loschko J Schlitzer A Dudziak D Drexler I Sandholzer N Bourquin C. Antigen delivery to plasmacytoid dendritic cells via BST2 induces protective T cell-mediated immunity. J Immunol. (2011) 186:6718–25. 10.4049/jimmunol.1004029 21555533

[130] Neubert K Lehmann CHK Heger L Baranska A Staedtler AM Buchholz VR. Antigen delivery to CD11c+CD8- dendritic cells induces protective immune responses against experimental melanoma in mice *in vivo*. J Immunol. (2014) 192:5830–8. 10.4049/jimmunol.1300975 24829411

[131] Lou Y Liu C Kim GJ Liu YJ Hwu P Wang G. Plasmacytoid dendritic cells synergize with myeloid dendritic cells in the induction of antigen-specific antitumor immune responses. J Immunol. (2007) 178:1534–41. 10.4049/jimmunol.178.3.1534 17237402

[132] Cohn L Chatterjee B Esselborn F Smed-Sörensen A Nakamura N Chalouni C. Antigen delivery to early endosomes eliminates the superiority of human blood BDCA3+ dendritic cells at cross presentation. J Exp Med. (2013) 210:1049–63. 10.1084/jem.20121251 23569326 PMC3646496

[133] Sandoval F Terme M Nizard M Badoual C Bureau M-F Freyburger L. Mucosal imprinting of vaccine-induced CD8^+^ T cells is crucial to inhibit the growth of mucosal tumors. Sci Transl Med. (2013) 5:172ra20. 10.1126/scitranslmed.3004888 23408053 PMC4086646

[134] Charalambous A Oks M Nchinda G Yamazaki S Steinman RM. Dendritic cell targeting of survivin protein in a xenogeneic form elicits strong CD4+ T cell immunity to mouse survivin. J Immunol. (2006) 177:8410–21. 10.4049/jimmunol.177.12.8410 17142738

[135] Kratky W Reis e Sousa C Oxenius A Spörri R. Direct activation of antigen-presenting cells is required for CD8^+^ T-cell priming and tumor vaccination. Proc Natl Acad Sci USA. (2011) 108:17414–9. 10.1073/pnas.1108945108 21987815 PMC3198339

[136] Yoshida S Shime H Takeda Y Nam JM Takashima K Matsumoto M. Toll-like receptor 3 signal augments radiation-induced tumor growth retardation in a murine model. Cancer Sci. (2018) 109:956–65. 10.1111/cas.13543 29465830 PMC5891207

[137] Zhou J Liu L Yang T Lu B. Prognostic and therapeutic value of CD103+ cells in renal cell carcinoma. Exp Ther Med. (2018) 15):4979–86. 10.3892/etm.2018.6025 29805521 PMC5952092

[138] Ngiow SF von Scheidt B Akiba H Yagita H Teng MWL Smyth MJ. Anti-TIM3 antibody promotes T cell IFN-γ-mediated antitumor immunity and suppresses established tumors. Cancer Res. (2011) 71:3540–51. 10.1158/0008-5472.CAN-11-0096 21430066

[139] Moynihan KD Opel CF Szeto GL Tzeng A Zhu EF Engreitz JM. Eradication of large established tumors in mice by combination immunotherapy that engages innate and adaptive immune responses. Nat Med. (2016) 22:1402–10. 10.1038/nm.4200 27775706 PMC5209798

[140] Nierkens S den Brok MH Roelofsen T Wagenaars JAL Figdor CG Ruers TJ. Route of administration of the TLR9 agonist CpG critically determines the efficacy of cancer immunotherapy in mice. PLoS ONE (2009) 4:e8368. 10.1371/journal.pone.0008368 20020049 PMC2791230

[141] Bald T Landsberg J Lopez-Ramos D Renn M Glodde N Jansen P. Immune cell–poor melanomas benefit from PD-1 blockade after targeted type I IFN activation. Cancer Discov. (2014) 4:674–87. 10.1158/2159-8290.CD-13-0458 24589924

[142] Corrales L Glickman LH McWhirter SM Kanne DB Sivick KE Katibah GE. Direct activation of STING in the tumor microenvironment leads to potent and systemic tumor regression and immunity. Cell Rep. (2015) 11:1018–30. 10.1016/j.celrep.2015.04.031 25959818 PMC4440852

[143] Cauwels A Van Lint S Garcin G Bultinck J Paul F Gerlo S. A safe and highly efficient tumor-targeted type I interferon immunotherapy depends on the tumor microenvironment. Oncoimmunology (2018) 7:e1398876. 10.1080/2162402x.2017.1398876 29399401 PMC5790344

[144] Mittal D Young A Stannard K Yong M Teng MWL Allard B. Antimetastatic effects of blocking PD-1 and the adenosine A2A receptor. Cancer Res. (2014) 74:3652–8. 10.1158/0008-5472.CAN-14-0957 24986517

[145] Le Mercier I Poujol D Sanlaville A Sisirak V Gobert M Durand I Tumor promotion by intratumoral plasmacytoid dendritic cells is reversed by TLR7 ligand treatment. Cancer Res. (2013) 73:4629–40. 10.1158/0008-5472.CAN-12-3058 23722543

[146] Georgoudaki A-M Prokopec KE Boura VF Hellqvist E Sohn S Östling J. Reprogramming tumor-associated macrophages by antibody targeting inhibits cancer progression and metastasis. Cell Rep. (2016) 15:2000–11. 10.1016/j.celrep.2016.04.084 27210762

[147] Balan S Arnold-Schrauf C Abbas A Couespel N Savoret J Imperatore F. Large-scale human dendritic cell differentiation revealing notch-dependent lineage bifurcation and heterogeneity. Cell Reports (2018) 24:1902–15.e6. 10.1016/j.celrep.2018.07.033 30110645 PMC6113934

[148] Kirkling ME Cytlak U Lau CM Lewis KL Resteu A Khodadadi-Jamayran A. Notch signaling facilitates *in vitro* generation of cross-presenting classical dendritic cells. Cell Rep. (2018) 23:3658–72.e6. 10.1016/j.celrep.2018.05.068 29925006 PMC6063084

[149] Crozat K Guiton R Guilliams M Henri S Baranek T Schwartz-Cornil I. Comparative genomics as a tool to reveal functional equivalences between human and mouse dendritic cell subsets. Immunol Rev. (2010) 234:177–98. 10.1111/j.0105-2896.2009.00868.x 20193019

[150] Haniffa M Shin A Bigley V McGovern N Teo P See P. Human tissues contain CD141hi cross-presenting dendritic cells with functional homology to mouse CD103+ nonlymphoid dendritic cells. Immunity (2012) 37:60–73. 10.1016/j.immuni.2012.04.012 22795876 PMC3476529

[151] Schlitzer A McGovern N Teo P Zelante T Atarashi K Low D. IRF4 transcription factor-dependent CD11b+ dendritic cells in human and mouse control mucosal IL-17 cytokine responses. Immunity (2013) 38:970–83. 10.1016/j.immuni.2013.04.011 23706669 PMC3666057

[152] Watchmaker PB Lahl K Lee M Baumjohann D Morton J Kim SJ. Comparative transcriptional and functional profiling defines conserved programs of intestinal DC differentiation in humans and mice. Nat Immunol. (2014) 15:98–108. 10.1038/ni.2768 24292363 PMC3942165

[153] Carpentier S Vu Manh T-P Chelbi R Henri S Malissen B Haniffa M. Comparative genomics analysis of mononuclear phagocyte subsets confirms homology between lymphoid tissue-resident and dermal XCR1(+) DCs in mouse and human and distinguishes them from Langerhans cells. J Immunol Methods (2016) 432:35–49. 10.1016/j.jim.2016.02.023 26966045 PMC4859332

[154] Bigley V Maisuria S Cytlak U Jardine L Care MA Green K. Biallelic interferon regulatory factor 8 mutation: a complex immunodeficiency syndrome with dendritic cell deficiency, monocytopenia, and immune dysregulation. J Aller Clin Immunol. (2018) 141:2234–48. 10.1016/j.jaci.2017.08.044 29128673 PMC5986711

[155] Segura E Durand M Amigorena S. Similar antigen cross-presentation capacity and phagocytic functions in all freshly isolated human lymphoid organ-resident dendritic cells. J Exp Med (2013) 210:1035–47. 10.1084/jem.20121103 23569327 PMC3646495

[156] Jongbloed SL Kassianos AJ McDonald KJ Clark GJ Ju X Angel CE. Human CD141+ (BDCA-3)+ dendritic cells (DCs) represent a unique myeloid DC subset that cross-presents necrotic cell antigens. J Exp Med. (2010) 207:1247–1260. 10.1084/jem.20092140 20479116 PMC2882828

[157] Chiang M-C Tullett KM Lee YS Idris A Ding Y McDonald KJ. Differential uptake and cross-presentation of soluble and necrotic cell antigen by human DC subsets. Eur J Immunol. (2016) 46:329–39. 10.1002/eji.201546023 26542182

[158] Sancho D Joffre OP Keller AM Rogers NC Martinez D Hernanz-Falcon P Rosewell I Reis e Sousa C. Identification of a dendritic cell receptor that couples sensing of necrosis to immunity. Nature (2009) 458:899–903. 10.1038/nature07750 19219027 PMC2671489

[159] Huysamen C Willment JA Dennehy KM Brown GD. CLEC9A is a novel activation C-type lectin-like receptor expressed on BDCA3+ dendritic cells and a subset of monocytes. J Biol Chem. (2008) 283:16693–701. 10.1074/jbc.M709923200 18408006 PMC2562446

[160] Caminschi I Proietto AI Ahmet F Kitsoulis S Shin Teh J Lo JCY. The dendritic cell subtype-restricted C-type lectin Clec9A is a target for vaccine enhancement. Blood (2008) 112:3264–73. 10.1182/blood-2008-05-155176 18669894 PMC2569177

[161] Lin ML Zhan Y Proietto AI Prato S Wu L Heath WR. Selective suicide of cross-presenting CD8^+^ dendritic cells by cytochrome c injection shows functional heterogeneity within this subset. Proc Natl Acad Sci USA. (2008) 105:3029–34. 10.1073/pnas.0712394105 18272486 PMC2268579

[162] Savina A Peres A Cebrian I Carmo N Moita C Hacohen N. The small GTPase Rac2 controls phagosomal alkalinization and antigen crosspresentation selectively in CD8(+) dendritic cells. Immunity (2009) 30:544–55. 10.1016/j.immuni.2009.01.013 19328020

[163] Nair-Gupta P Baccarini A Tung N Seyffer F Florey O Huang Y. TLR signals induce phagosomal MHC-I delivery from the endosomal recycling compartment to allow cross-presentation. Cell (2014) 158:506–21. 10.1016/j.cell.2014.04.054 25083866 PMC4212008

[164] Kretzer NM Theisen DJ Tussiwand R Briseño CG Grajales-Reyes GE Wu X. RAB43 facilitates cross-presentation of cell-associated antigens by CD8α+ dendritic cells. J Exp Med. (2016) 213:2871–83. 10.1084/jem.20160597 27899443 PMC5154939

[165] Crozat K Tamoutounour S Vu Manh T-P Fossum E Luche H Ardouin L. Cutting edge: expression of XCR1 defines mouse lymphoid-tissue resident and migratory dendritic cells of the CD8α+ type. J Immunol. (2011) 187:4411–5. 10.4049/jimmunol.1101717 21948982

[166] Galibert L Diemer GS Liu Z Johnson RS Smith JL Walzer T. Nectin-like protein 2 defines a subset of T-cell zone dendritic cells and is a ligand for class-I-restricted T-cell-associated molecule. J Biol Chem. (2005) 280:21955–64. 10.1074/jbc.M502095200 15781451

[167] Nizzoli G Krietsch J Weick A Steinfelder S Facciotti F Gruarin P. Human CD1c+ dendritic cells secrete high levels of IL-12 and potently prime cytotoxic T-cell responses. Blood (2013) 122:932–42. 10.1182/blood-2013-04-495424 23794066

[168] Hémont C Neel A Heslan M Braudeau C Josien R. Human blood mDC subsets exhibit distinct TLR repertoire and responsiveness. J Leukoc Biol. (2013) 93:599–609. 10.1189/jlb.0912452 23341538

[169] Lee J Breton G Oliveira TYK Zhou YJ Aljoufi A Puhr S. Restricted dendritic cell and monocyte progenitors in human cord blood and bone marrow. J Exp Med. (2015) 212:385–399. 10.1084/jem.20141442 25687283 PMC4354373

[170] Lauterbach H Bathke B Gilles S Traidl-Hoffmann C Luber CA Fejer G. Mouse CD8alpha+ DCs and human BDCA3+ DCs are major producers of IFN-lambda in response to poly IC. J Exp Med. (2010) 207:2703–17. 10.1084/jem.20092720 20975040 PMC2989774

[171] Yarovinsky F Zhang D Andersen JF Bannenberg GL Serhan CN Hayden MS. TLR11 activation of dendritic cells by a protozoan profilin-like protein. Science (2005) 308:1626–9. 10.1126/science.1109893 15860593

[172] Raetz M Kibardin A Sturge CR Pifer R Li H Burstein E. Cooperation of TLR12 and TLR11 in the IRF8-dependent IL-12 response to Toxoplasma gondii profilin. J Immunol. (2013) 191:4818–27. 10.4049/jimmunol.1301301 24078692 PMC3805684

[173] Poulin LF Salio M Griessinger E Anjos-Afonso F Craciun L Chen J-L. Characterization of human DNGR-1+ BDCA3+ leukocytes as putative equivalents of mouse CD8alpha+ dendritic cells. J Exp Med. (2010) 207:1261–71. 10.1084/jem.20092618 20479117 PMC2882845

[174] Sittig SP Bakdash G Weiden J Sköld AE Tel J Figdor CG. A comparative study of the T cell stimulatory and polarizing capacity of human primary blood dendritic cell subsets. Med Inflamm. (2016) 2016:3605643. 10.1155/2016/3605643 27057096 PMC4761397

[175] Mittag D Proietto AI Loudovaris T Mannering SI Vremec D Shortman K. Human dendritic cell subsets from spleen and blood are similar in phenotype and function but modified by donor health status. J Immunol. (2011) 186:6207–17. 10.4049/jimmunol.1002632 21515786

[176] Granot T Senda T Carpenter DJ Matsuoka N Weiner J Gordon CL. Dendritic cells display subset and tissue-specific maturation dynamics over human life. Immunity (2017) 46:504–15. 10.1016/j.immuni.2017.02.019 28329707 PMC5415308

[177] Gentles AJ Newman AM Liu CL Bratman SV Feng W Kim D. The prognostic landscape of genes and infiltrating immune cells across human cancers. Nat Med. (2015) 21:938–45. 10.1038/nm.3909 26193342 PMC4852857

[178] Becht E Giraldo NA Lacroix L Buttard B Elarouci N Petitprez F Estimating the population abundance of tissue-infiltrating immune and stromal cell populations using gene expression. Genome Biol. (2016) 17:218 10.1186/s13059-016-1070-5 27765066 PMC5073889

[179] Li B Severson E Pignon J-C Zhao H Li T Novak J. Comprehensive analyses of tumor immunity: implications for cancer immunotherapy. Genome Biol. (2016) 17:174. 10.1186/s13059-016-1028-7 27549193 PMC4993001

[180] Charoentong P Finotello F Angelova M Mayer C Efremova M Rieder D. Pan-cancer immunogenomic analyses reveal genotype-immunophenotype relationships and predictors of response to checkpoint blockade. Cell Rep. (2017) 18:248–62. 10.1016/j.celrep.2016.12.019 28052254

[181] Schelker M Feau S Du J Ranu N Klipp E MacBeath G. Estimation of immune cell content in tumour tissue using single-cell RNA-seq data. Nat Commun. (2017) 8:2032. 10.1038/s41467-017-02289-3 29230012 PMC5725570

[182] Michea P Noël F Zakine E Czerwinska U Sirven P Abouzid O. Adjustment of dendritic cells to the breast-cancer microenvironment is subset specific. Nat Immunol. (2018) 19:885–97. 10.1038/s41590-018-0145-8 30013147

[183] Györffy B Lanczky A Eklund AC Denkert C Budczies J Li Q. An online survival analysis tool to rapidly assess the effect of 22,277 genes on breast cancer prognosis using microarray data of 1,809 patients. Breast Cancer Res Treat. (2010) 123:725–31. 10.1007/s10549-009-0674-9 20020197

[184] Bogunovic D O'Neill DW Belitskaya-Levy I Vacic V Yu Y-L Adams S. Immune profile and mitotic index of metastatic melanoma lesions enhance clinical staging in predicting patient survival. Proc Natl Acad Sci USA. (2009) 106:20429–34. 10.1073/pnas.0905139106 19915147 PMC2787158

[185] Lavin Y Kobayashi S Leader A Amir ED Elefant N Bigenwald C. Innate immune landscape in early lung adenocarcinoma by paired single-cell analyses. Cell (2017) 169:750–765 e17. 10.1016/j.cell.2017.04.014 28475900 PMC5737939

[186] Sluijter BJ van den Hout MF Koster BD van Leeuwen PA Schneiders FL van de Ven R. Arming the melanoma sentinel lymph node through local administration of CpG-B and GM-CSF: recruitment and activation of BDCA3/CD141(+) dendritic cells and enhanced cross-presentation. Cancer Immunol Res. (2015) 3:495–505. 10.1158/2326-6066.CIR-14-0165 25633713

[187] van den Hout MFCM Sluijter BJR Santegoets SJAM van Leeuwen PAM van den Tol MP van den Eertwegh AJM Local delivery of CpG-B and GM-CSF induces concerted activation of effector and regulatory T cells in the human melanoma sentinel lymph node. Cancer Immunol Immunother. (2016) 65:405–15. 10.1007/s00262-016-1811-z 26935057 PMC4826413

[188] Bhardwaj N Treatment of Solid Tumors With Intratumoral Hiltonol® (Poly-ICLC)-Full Text View - ClinicalTrials.gov. Available online at: https://clinicaltrials.gov/ct2/show/NCT01984892 (Accessed Dec 9, 2018).

[189] Saxena M Bhardwaj N. Re-emergence of dendritic cell vaccines for cancer treatment. Trends Can. (2018) 4:119–37. 10.1016/j.trecan.2017.12.007 29458962 PMC5823288

[190] Dhodapkar MV Sznol M Zhao B Wang D Carvajal RD Keohan ML. Induction of antigen-specific immunity with a vaccine targeting NY-ESO-1 to the dendritic cell receptor DEC-205. Sci Transl Med. (2014) 6:232ra51. 10.1126/scitranslmed.3008068 24739759 PMC6151129

[191] Tel J Aarntzen EHJG Baba T Schreibelt G Schulte BM Benitez-Ribas D. Natural human plasmacytoid dendritic cells induce antigen-specific T-cell responses in melanoma patients. Cancer Res. (2013) 73:1063–75. 10.1158/0008-5472.CAN-12-2583 23345163

[192] Schreibelt G Bol KF Westdorp H Wimmers F Aarntzen EHJG Duiveman-de Boer T. Effective clinical responses in metastatic melanoma patients after vaccination with primary myeloid dendritic cells. Clin Cancer Res. (2016) 22:2155–66. 10.1158/1078-0432.CCR-15-2205 26712687

[193] Terhorst D Fossum E Baranska A Tamoutounour S Malosse C Garbani M. Laser-assisted intradermal delivery of adjuvant-free vaccines targeting XCR1+ dendritic cells induces potent antitumoral responses. J Immunol. (2015) 194:5895–902. 10.4049/jimmunol.1500564 25941327

[194] Yan Z Wu Y Du J Li G Wang S Cao W. A novel peptide targeting Clec9a on dendritic cell for cancer immunotherapy. Oncotarget (2016) 7:40437–50. 10.18632/oncotarget.9624 27250027 PMC5130018

[195] Pearson FE Chang K Minoda Y Rojas IML Haigh OL Daraj G. Activation of human CD141+ and CD1c+ dendritic cells *in vivo* with combined TLR3 and TLR7/8 ligation. Immunol Cell Biol. (2018) 96:390–400. 10.1111/imcb.12009 29344995

[196] Deauvieau F Ollion V Doffin A-C Achard C Fonteneau J-F Verronese E. Human natural killer cells promote cross-presentation of tumor cell-derived antigens by dendritic cells. Int J Can. (2015) 136:1085–94. 10.1002/ijc.29087 25046660

[197] Saxena M Balan S Roudko V Bhardwaj N. Towards superior dendritic-cell vaccines for cancer therapy. Nat Biomed Eng. (2018) 2:341–6. 10.1038/s41551-018-0250-x 30116654 PMC6089533

[198] Wang B Zaidi N He L-Z Zhang L Kuroiwa JM Keler T. Targeting of the non-mutated tumor antigen HER2/neu to mature dendritic cells induces an integrated immune response that protects against breast cancer in mice. Breast Cancer Res. (2012) 14:R39. 10.1186/bcr3135 22397502 PMC3446373

[199] Mahnke K Qian Y Fondel S Brueck J Becker C Enk AH. Targeting of antigens to activated dendritic cells *in vivo* cures metastatic melanoma in mice. Cancer Res. (2005) 65:7007–12. 10.1158/0008-5472.CAN-05-0938 16061687

[200] Hartung E Becker M Bachem A Reeg N Jäkel A Hutloff A Induction of potent CD8 T cell cytotoxicity by specific targeting of antigen to cross-presenting dendritic cells *in vivo* via murine or human XCR1. J Immunol. (2015) 194:1069–79. 10.4049/jimmunol.1401903 25520399

[201] Sancho D Mourão-Sá D Joffre OP Schulz O Rogers NC Pennington DJ. Tumor therapy in mice via antigen targeting to a novel, DC-restricted C-type lectin. J Clin Investig. (2008) 118:2098–110. 10.1172/JCI34584 18497879 PMC2391066

[202] Sartorius R Bettua C D'Apice L Caivano A Trovato M Russo D. Vaccination with filamentous bacteriophages targeting DEC-205 induces DC maturation and potent anti-tumor T-cell responses in the absence of adjuvants. Eur J Immunol. (2011) 41:2573–84. 10.1002/eji.201141526 21688262

[203] Broekhoven CL van Parish CR Demangel C Britton WJ Altin JG. Targeting dendritic cells with antigen-containing liposomes: a highly effective procedure for induction of antitumor immunity and for tumor immunotherapy. Cancer Res. (2004) 64:4357–65. 10.1158/0008-5472.CAN-04-0138 15205352

[204] Picco G Beatson R Taylor-Papadimitriou J Burchell JM. Targeting DNGR-1 (CLEC9A) with antibody/MUC1 peptide conjugates as a vaccine for carcinomas. Euro J Immunol. (2014) 44:1947–55. 10.1002/eji.201344076 24648154 PMC4209794

[205] Kreutz M Giquel B Hu Q Abuknesha R Uematsu S Akira S. Antibody-antigen-adjuvant conjugates enable co-delivery of antigen and adjuvant to dendritic cells in cis but only have partial targeting specificity. PLoS ONE (2012) 7:e40208. 10.1371/journal.pone.0040208 22808118 PMC3393736

[206] Flacher V Tripp CH Mairhofer DG Steinman RM Stoitzner P Idoyaga J. Murine Langerin+ dermal dendritic cells prime CD8^+^ T cells while Langerhans cells induce cross-tolerance. EMBO Mol Med. (2014) 6:1191–204. 10.15252/emmm.201303283 25085878 PMC4197865

[207] Bonifaz LC Bonnyay DP Charalambous A Darguste DI Fujii S-I Soares H. *In vivo* targeting of antigens to maturing dendritic cells via the DEC-205 receptor improves T cell vaccination. J Exp Med. (2004) 199:815–24. 10.1084/jem.20032220 15024047 PMC2212731

