# Supplementary material for: Are Conventional Type 1 Dendritic Cells Critical for Protective Antitumor Immunity and How?
Source: Front Immunol. 2019 Feb 12;10:9. doi: 10.3389/fimmu.2019.00009 (PMC6379659; doi:10.3389/fimmu.2019.00009)
Supplement: Supplementary file 1 [file Table_1.docx]

Supplementary Material

Are conventional type 1 dendritic cells critical for protective anti-tumoral immunity and how?

Jean-Charles Cancel^†^, Karine Crozat^†*^, Marc Dalod^†^*, Raphaël Mattiuz^†^

^†^ All authors equally contributed to this work.

*** Correspondence:**Karine Crozat, [crozat@ciml.univ-mrs.fr](mailto:crozat@ciml.univ-mrs.fr) and Marc Dalod [dalod@ciml.univ-mrs.fr](mailto:dalod@ciml.univ-mrs.fr)

# Supplementary Tables

**Table S1: Mobilizing specifically cDC1 functions through cDC1 marker targeting**

(see below).

| **Targeted cDC1 function** | **Targeted marker** | **Vaccine form** | **Tumor model** | **Prophylactic effect and time of administration before tumor** | **Therapeutic effect and time of administration after tumor** | **Period of follow up of tumor growth** | **Ref.** |
| --- | --- | --- | --- | --- | --- | --- | --- |
| **Tumor Ag targeting** | Clec9a | gp100, TRP-1 and TRP-2 coupled anti-Clec9a + antiCD40 +/- poly(I:C) | B16-OVA metastasis | Less metastasis (30d) | Less metastasis  (3d) | < 18 d | (201) |
|  |  | MUC-1-derived LLLL and ALG peptides coupled to antiClec9a + antiCD40 and poly(I:C) | MUC1 and A2K/b expressing colon cancer cells MC38 in MUC1;A2K/b Tg mice | Short delay in tumor growth (37d + 22d + 7d) | Low delayed tumor growth (4d + 14d) | < 35 d | (204) |
|  |  | OVA-coencapsulated in NP/antiClec9a  + αGalCer | OVA expressing E.G7 thymoma s.c. | Delayed tumor growth  (7d) | Delayed tumor growth in OT-I reconstituted mice  (5d) | < 15 d | (120) |
|  |  | TRP2 and gp100-coencapsulated in NP/ antiClec9a + αGalCer | B16-F10 melanoma | Significant delayed of tumor growth (7d) | Delayed tumor growth (5d + 12d) | < 20 d | (120) |
|  | XCR1 | XCL1-OVA vaccibody, or OVA coupled anti-XCR1 + LPS | OVA expressing E.G7 thymoma | Decreased tumor mass (7d) | n.d. | < 14 d | (134) |
|  |  | XCL1-OVA vaccibody + epithelial barrier disruption | B16-OVA melanoma | Transient protection (30d) | Delayed tumor growth (3d) | < 16 d | (193) |
|  | DEC-205 | OVA coupled antiDEC205 + antiCD40 | B16-OVA melanoma | Transient protection (60d) | Transient protection (7d) | < 19 d | (207) |
|  |  | OVA coupled antiDEC205 + antiCD40 and poly(I:C) | B16-OVA melanoma | Significant protection (60d + OVA boost at 39d) | Delayed tumor growth (5d) | < 29 d | (130) |
|  |  | OVA coupled antiDEC205 + imiquimod | B16-OVA melanoma | Delayed tumor growth (30d) | Delayed tumor growth (7d) | < 20 d | (206) |
|  |  | HER2 coupled antiDEC205 + poly(I:C) | NT2.5 mammary tumor (FVB/N) | Transient protection (10d) | n.d. | < 30 d | (198) |
|  |  | mSurvinin coupled antiDEC205 + antiCD40 and poly(I:C) | A20 lymphoma (BALB/c) | No effect | n.d. | n.a. | (134) |
|  |  | OVA-coupled antiDEC205 + CpG | B16-OVA metastasis | Protection depends on Ag amount (30d) | No effect on metastasis numbers but on their size (6d) | < 18 d | (205) |
|  |  | TRP-coupled antiDEC205 + CpG | B16-OVA metastasis | Significant protection (7d) | n.d. | < 14 d | (199) |
|  |  | TRP and gp100 coupled antiDEC205 + CpG | B16-F10 melanoma | n.d. | Significant delayed tumor growth  (0 + 6d) | < 42 d | (199) |
|  |  | OVA-coencapsulated in NP/ antiDEC205 + αGalCer | B16-OVA melanoma | Delayed tumor growth (7d) | n.d. | < 20 d | (121) |
|  |  | DNA vaccine : HER2 fused to antiDEC205 ScFv | D2F2 mammary tumor (BALB/c) | Total protection (21d + 7d) | Significant delayed tumor growth (7d + 21d)  Effect significantly improved when combined with chemotherapy | > 55 d | (124) |
|  |  | DNA vaccine : Neu ectodomain fused to antiDEC205 ScFv | Spontaneous mammary tumor (BALB/NeuT) | Delayed carcinogenesis (8w + 10w after birth) | Total protection (8w + 10w after birth, and when combined to chemotherapy) | > 55 d | (124) |
|  |  | Adenoviral vectors encoding a DEC205 targeted OVA | B16-OVA melanoma | Partial effect on tumor growth (36d) | n.d. | < 25 d | (125) |
|  | Langerin | OVA coupled antiLangerin + imiquimod | B16-OVA melanoma | No significant effect (30d) | n.d. | < 20 d | (206) |
| **Targeted tumor Ag and adjuvant delivery** | Clec9a | OVA-encapsuled in a Clec9a targeted oil in water nanoemulsion | orthotopic PyMT-mCherry-OVA mammary tumor | n.d. | Low delayed tumor growth (14d) | < 24 d | (127) |
|  |  | E6/E7-encapsuled in a WH or F-Actin oil in water nanoemulsion | TC1 HPV-related cancer model s.c. | n.d. | Delayed tumor growth (14d + 21d + 28d + 35d) | < 42 d | (127) |
|  |  | neoAg-encapsuled in a WH oil in water nanoemulsion | B16-F10 melanoma | n.d. | Delayed tumor growth (8d + 15d + 22d + 29d) | < 60 d | (127) |
|  | DEC-205 | IFN-γ or LPS and OVA-encapsuled in anti-DEC205 ScFv Ab associated to stealth liposomes | B16-OVA metastasis | Significant control (28d + 21d +14d) | n.d. | < 16 d | (203) |
|  |  | Filamentous bacteriophage virions bearing OVA-coupled to antiDEC-205 ScFv | B16-OVA melanoma | Significant control (17d + 7d) | n.d. | < 40 d | (202) |
|  |  | PMV-associated anti-DEC205 ScFv containing IFN-γ or LPS | B16-OVA metastasis | Significant control (28d + 21d +14d) | Complete control with IFN-γ  (d3 + d6 + d9) | < 8 months | (203) |
| **Targeted induction of immunogenicity** | Clec9a | Delivery of a modified IFNα2 to cDC1s (anti-Clec9a-AcTaferon) +/- mAIM, chemotherapy or TNF-based therapy | B16 4T1 mammary, A20 (BALB/C) | n.d. | Significant control (6 to 8 consecutive injections starting at 7d) potentiates the effect of chemotherapy, cytokine therapy and of ICB associated with Treg depletion | < 22 d | (115) |

All tumor models were engrafted in *C57BL*/6J unless otherwise specified. B16-OVA and B16-F10 were all s.c. inoculated unless otherwise specified. ScFv: single chain full-length variable Ab fragments; n.d. not determined; PMV, Plasma Membrane Vesicles; WH, peptide binding Clec9a; HPV, Human Papilloma Virus.
